# π–π-Induced aggregation and single-crystal fluorescence anisotropy of 5,6,10b-tri­aza­acephenanthrylene

**DOI:** 10.1107/S2052252518001987

**Published:** 2018-04-18

**Authors:** Katarzyna Ostrowska, Davide Ceresoli, Katarzyna Stadnicka, Marlena Gryl, Marco Cazzaniga, Raffaella Soave, Bogdan Musielak, Łukasz J. Witek, Piotr Goszczycki, Jarosław Grolik, Andrzej M. Turek

**Affiliations:** a Faculty of Chemistry, Jagiellonian University, Gronostajowa 2, Kraków, Małopolskie 30-387, Poland; b Istituto di Scienze e Tecnologie Molecolari, CNR-ISTM and INSTM UdR di Milano, via C. Golgi, Milano, 20133, Italy

**Keywords:** optical materials, fluorescence, anisotropy, JH aggregates, tri­aza­acephenan­thrylene (TAAP), hydrogen bonding, π–π interactions, transition dipole moments

## Abstract

The structural origin of absorption and fluorescence anisotropy of the single crystal of the π-conjugated heterocyclic system 5,6,10b-tri­aza­acephenan­thrylene, TAAP, is presented in this study. X-ray analysis shows that the crystal framework in the space group *P*  is formed by centrosymmetric dimers of face-to-face mutually oriented TAAP molecules joined by π–π non-covalent interactions. The conformation of the TAAP molecule is stabilized by intramolecular C—H⋯N(*sp* ^2^), N(*sp* ^2^)H⋯π(CN), and C—H⋯O(*sp* ^2^) hydrogen bonds. The presence of weak π–π interactions is confirmed by quantum theory of atoms in molecules (QTAIM) and non-covalent interaction (NCI) analysis. The analysis of the optical spectra of TAAP in solution and in the solid state does not allow the specification of the aggregation type. DFT calculations for the dimer in the gas phase indicate that the lowest singlet excitation is forbidden by symmetry, suggesting H-type aggregation, even though the overall absorption spectrum is bathochromically shifted as for the J-type. The experimental determination of the permanent dipole moment of a TAAP molecule in 1,4-dioxane solution indicates the presence of the monomer form. The calculated absorption and emission spectra of the crystal in a simple approximation are consistent with the experimentally determined orientation of the absorption and emission transition dipole moments in TAAP single crystals. The electrostatic interaction between monomers with a permanent dipole moment (*ca* 4 D each) could result in the unusual spectroscopic JH-aggregate behaviour of the TAAP dimer.

## Abstract 2

The influence of π–π overlap in centrosymmetric dimers on the aggregation type, single-crystal absorption and fluorescence anisotropy of the new heterocyclic system 5,6,10b-tri­aza­acephenanthrylene is presented.

## 1. Introduction   

**Figure 1:**
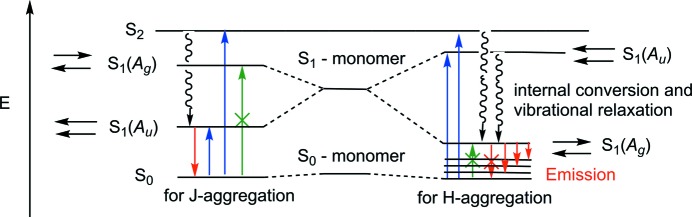
Jablonski diagram for H- and J-type aggregates of the π-dimer with a centre of inversion (allowed absorption in blue, forbidden absorption in green and emission in red).

Optical properties of π-conjugated crystalline materials depend on the packing arrangement in the crystal structure (Varghese & Das, 2011 ▸; Varughese, 2014 ▸). The molecular self-assembly is guided by hydrogen-bonding, π–π stacking and C—H⋯π non-covalent interactions between planar π-conjugated systems (Loots & Barbour, 2012 ▸). There are three main geometries of aromatic π–π interactions in the dimer that are known, taking into account the relative position of the planar aromatic molecules: edge-to-face (T-shaped), eclipsed face-to-face (cofacial, sandwich-type) and offset face-to-face (slipped, head-to-tail) (Loots & Barbour, 2012 ▸; Janiak, 2000 ▸; Hunter *et al.*, 2001 ▸). The strength of electronic transitions for π–π coupled conjugated systems depends on the vector sum of the transition dipole moments for monomers. There are two main types of aggregates, J-type (Würthner *et al.*, 2011 ▸) and H-type (Nüesch & Grätzel, 1995 ▸; Spano, 2000 ▸; Xu *et al.*, 2012 ▸), that are considered by Kasha’s approach (Gierschner & Park, 2013 ▸; McRae & Kasha, 1958 ▸; Kasha *et al.*, 1965 ▸). For J-type aggregates, the π-dimers consist of coplanar mutually shifted molecules with a head-to-tail orientation of the monomer transition dipole moments. In this case, the excited state of the dimer splits into two states, a lower energy state and a higher energy state, which have parallel and antiparallel transition dipole moments, respectively. The absorption and emission transitions gain only the lower state with a non-zero total transition dipole moment. The absorption band for the dimer related to the *S*_0_→*S*_1_ transition is bathochromically shifted with respect to that of the monomer band. For H-type aggregates, the molecules are aligned face-to-face, with an antiparallel orientation of the molecular transition dipole moments. The excited π-dimer state splits into a higher state and a lower state, with a parallel and an antiparallel orientation of the transition dipole moments, respectively (Fig. 1 ▸). The absorption transitions to the higher state are not forbidden for H-type aggregates and, as a result, a hypsochromically shifted absorption band is usually observed. The emission proceeds from the lowest vibrational level of the lower-energy H-dimer excited state to the weakly allowed higher vibrational states of the ground electronic state (Spano, 2010 ▸; Spano & Silva, 2014 ▸). As a result of fast non-radiative relaxation, the weak emission implies that H-type aggregates are considered as poor emitters (Fang *et al.*, 2014 ▸).

The determination of the aggregation type relying only on blue or red spectral band shifts from the monomer in solution to the dimer in the solid state may be misleading for several reasons. Kasha’s exciton theory explains the shifts of absorption bands for *loosely-bonded dimers* (Gierschner & Park, 2013 ▸; McRae & Kasha, 1958 ▸; Kasha *et al.*, 1965 ▸). Molecules with permanent dipole moments may induce both intra- and intermolecular charge transfer, resulting in bathochromic spectral band shifts (Huang *et al.*, 2011 ▸). Enhanced *intrachain planarization*, as in the case of poly(3-hexyl­thio­phene) aggregates (Brown *et al.*, 2003 ▸), may also lead to red shifts with the H-type geometry and nature. Crystals that revealed such unusual spectral characteristics are classified as HJ- or JH-type according to the new paradigm for organic electronic materials design (Yamagata *et al.*, 2014 ▸). In this two-letter notation, the first refers to the exciton Coulombic long-range coupling and the second to the exciton charge transfer (CT) short-range coupling. These couplings compete with the π–π interactions between neighbouring molecules (Yamagata *et al.*, 2014 ▸). Coulombic coupling induces H-type aggregation behaviour with the exciton coupling parameter *J*_0_ > 0, whereas CT coupling promotes J-type with *J*_0_ < 0 (Spano & Silva, 2014 ▸; Yamagata *et al.*, 2014 ▸).

**Figure 2:**
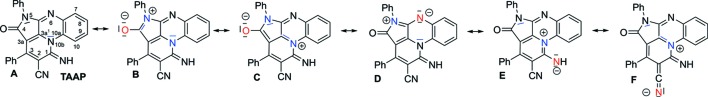
Zwitterionic resonance structures of TAAP.

Recently, we have described the synthesis of the new π-conjugated heterocyclic system 5,6,10b-tri­aza­acephenanthrylene (TAAP) (Ostrowska *et al.*, 2016 ▸), which is structurally related to the naturally occurring alkaloids, such as *aristolactams* (Kumar *et al.*, 2003 ▸; Michl *et al.*, 2014 ▸). The π-conjugated TAAP planar chromophore exhibits a donor–acceptor architecture with a few different acceptor groups: carbonyl imide, nitrile, and two imines. This specific electronic arrangement results in delocalization of the N5 and N10b lone electron pairs into the π system, leading to the formation of zwitterions. The possible resonance structures are shown in Fig. 2 ▸. In the **B** and **C** forms, the carbonyl O atom withdraws the electron lone pair from either the N5 or N10b atoms. In the **D** form, the electron lone pair from N5 is shifted to atom N6, whereas in the **E** and **F** forms, the electron lone pair of N10b is attracted by the imine N atom at C1 and the nitrile N atom at C2, respectively. It was shown that pyrazine-fused heterocyclic systems with two imine moieties can be used as electron-withdrawing units to construct conjugated organic materials for optoelectronic applications (Lu *et al.*, 2014 ▸). In the TAAP heterocyclic system, the lone electron pair located at the *p* orbital of the N10b atom changes the character of the imine group from electron acceptor (typically observed for quinoxaline) to electron donor.

In the present study, we show the influence of face-to-face monomer π–π overlap in dimers on the aggregation type, single-crystal absorption and fluorescence anisotropy of the new heterocyclic system of TAAP. We have attempted to define the aggregation type of TAAP in order to understand its optical properties in solution and the crystalline state.

It should be noted that, until now, surprisingly little has been published on single-crystal fluorescence anisotropy, except for rubrene (El Helou *et al.*, 2010 ▸; Ma *et al.*, 2013 ▸), green fluorescent protein (GFP) (Inoué *et al.*, 2002 ▸; Rosell & Boxer, 2003 ▸) and complexes of nickel with Schiff bases (Hara *et al.*, 2011 ▸). However, it should be noted that recent publications concerning the usage of distinct fluorescence bands, based on intramolecular proton transfer, *e.g.* for alcohol vapour detection (Chen *et al.*, 2016 ▸), as well as the multicolour fluorescence of hetero-Ln-MOF (where MOF denotes metal–organic framework), hierarchical single crystals, provide potential applications in miniaturized optoelectronic devices (Pan *et al.*, 2017 ▸).

## 2. Experimental   

### 2.1. General aspects   

^1^H NMR and ^13^C NMR spectra were recorded using a Bruker Avance III 600 and a Bruker Avance II 300 at 300 K. The chemical shifts (*δ*) are reported in parts per million (ppm) on a *δ* scale downfield from TMS. The ^1^H NMR spectra were referenced internally to the residual proton resonance in CDCl_3_ (*δ* 7.26 ppm), CD_2_Cl_2_ (5.32 ppm), tetra­chloro­ethane-*d*_2_ (6.00 ppm), THF-*d*_8_ (1.73, 3.58 ppm), dioxane-*d*_8_ (3.53 ppm), toluene-*d*_8_ (7.09, 7.00, 6.98, 2.09 ppm) and benzene-*d*_6_ (*δ* 7.15 ppm). The fluorescence measurements for crystalline TAAP were recorded at 298 K with an excitation slit width of 5 nm, an emission slit of 5 nm and a 700 V PMT voltage. Sample crystals were illuminated under a polarized microscope with an illuminator using 8 pieces UV (410 nm) LED (LEDs were distributed around the sample every 45°) and observed through an analyzer (linear polarization filter). The UV–Vis spectra for TAAP were recorded using a Hitachi U-3900 H spectrophotometer in 1 cm cells at 298 K after equilibrating for 20 min.

The fluorescence measurements for TAAP solution were performed using a Hitachi F-4500 spectro­fluoro­meter. All spectra were recorded at 298 K with an excitation slit width of 5 nm, an emission slit of 5 nm and a 400 V or 600 V of the PMT voltage.

The fluorescence properties of a TAAP single crystal was analyzed with an Al–Si Nikon Inc. (Japan) confocal laser scanning system (LSCM) built onto a Nikon inverted Ti-E microscope using a Zeiss Plan-APOCHROMAT 100 ×/1.4na Oil DIC objective. Images were acquired at a resolution of 2048 × 2048. The Al–Si system was equipped with four-channel detection, as well as LSBF imaging by diascopic detection of forward scattered excitation laser light during confocal laser scanning. The excitation for confocal microscopy was provided by a diode laser with an excitation wavelength at 405 nm. The fluorescence spectra were collected using a 32-channel spectral detector.

### 2.2. Synthesis and growth of single crystals of TAAP   

The synthesis of TAAP has been described previously (Ostrowska *et al.*, 2016 ▸). The single crystals were isolated independently from highly concentrated solutions of TAAP in aceto­nitrile, aceto­nitrile–water, toluene, CDCl_3_, DMSO-*d*_6_, tetra­chloro­ethane-*d*_2_, THF-*d*_8_ and dioxane-*d*_8_. Natural cooling and slow evaporation of solvents in each case gave single crystals with the same habit and crystal structure.

### 2.3. X-ray crystallography   

The single-crystal X-ray diffraction experiments for TAAP_RT and TAAP_LT were carried out using monochromated Cu *K*α radiation (λ = 1.54178 Å) and Mo *K*α radiation (λ = 0.71073 Å), respectively. The experimental data were processed with *CrysAlisPro*. Crystallographic data and the details of data collection and crystal structure refinement are summarized in Table S1.1 (see supporting information). The structure was solved using direct methods with *SIR92* (Altomare *et al.*, 1994 ▸). A refinement procedure by full-matrix least-squares methods based on *F* ^2^ values against unique reflections, including all atomic fractional coordinates and anisotropic displacement parameters for non-H atoms, was performed using *SHELXL*2013 (Sheldrick, 2015 ▸). H atoms were found from difference Fourier maps and were included in the refinement procedure in the riding model, assuming isotropic displacement parameters.

## 3. Results and discussion   

### 3.1. X-ray crystal structure determination of TAAP   

**Figure 3:**
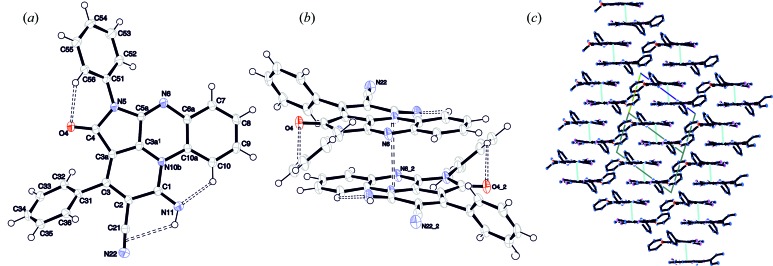
The molecular structure of TAAP (*a*) with the relevant atom-numbering scheme. The non-hydrogen atoms are represented as displacement ellipsoids plotted at the 30% probability level. (*b*) Side-by-side arrangement of two neighbouring chromophores with an interplanar distance of 3.413 Å. Intramolecular hydrogen bonds and the gravity centre distance are marked by dashed lines. (*c*) Packing scheme along [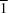
10]. Gravity-centre-to-gravity-centre distance for planar chromophores in the dimer is marked by a turquoize line. H atoms have been omitted for clarity and the unit cell is marked by a black line.

Single-crystal X-ray diffraction analysis revealed that TAAP crystallizes from most of the solvents such as THF, CHCl_3_, toluene, CH_3_CN, CH_3_CN–water mixture and tetra­chloro­ethane-*d*_2_, as triclinic crystals, with a centrosymmetric crystal structure in the space group *P* 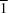 (Fig. 3 ▸ and Table S1.1). Selected geometrical parameters for TAAP_LT and TAAP_RT are summarized in Tables S1.2 and S1.3, respectively. The TAAP molecules form dimers arranged in a cofacial sandwich-like fashion, with an interlayer distance of 3.468 Å at 293 K (RT) and 3.413 Å at 130 K (LT).

The imine atom N11 forms two intramolecular interactions, acting as a strong donor for the weak C≡N π-acceptor (Desiraju & Steiner, 2001 ▸; Kumar *et al.*, 1998 ▸) [N11—H11⋯π(C≡N): 2.54 (2), 3.127 (2) Å, ∠DHA 124 (2)°] and strong acceptor for weak C—H donor [C10—H10⋯N11: 2.16, 2.795 (2) Å, ∠DHA 123°]. The nitrogen N11 atom is directly involved in the planarization of the antiaromatic heterocyclic system (Figs. 3 ▸ and S1.1–S1.3). The intramolecular hydrogen bond H10⋯N11 leads to the formation of an additional quasi-aromatic ring (Jezierska-Mazzarello *et al.*, 2012 ▸; Krygowski *et al.*, 2014 ▸) with the closed-coupled H11 proton and *sp* ^2^ hybridization of the N10*B* atom. That is confirmed by the valence angle sum of 360° at N10*B* (C3*A* ^1^—N10*B*—C10*A* = 116.9, C10*A*—N10*B*—C1 = 126.4 and C1—N10*B*—C3*A* ^1^ = 116.7°). Extension of the π-conjugation onto the imine and nitrile groups shifts the centre of gravity of the monomer towards atom N10*B* (Fig. 3 ▸ *b*). The two molecules of the dimer in the antiparallel configuration are shifted towards each other, with a centroid-to-centroid distance of 3.559 Å (the positions of centroids are defined for moieties without the imine and nitrile groups), resembling the π–π stacking interactions between adjacent sheets in graphite. The conformation of the TAAP molecule is additionally stabilized by the intramolecular hydrogen bond H56⋯O4 [C56—H56⋯O4: 2.56, 2.955 (2) Å, ∠DHA 105°] (Figs. S1.1 and S1.2, and Tables S1.4 and S1.5). The packing arrangement of the dimers is governed exclusively by very weak C—H⋯π and van der Waals interactions (Tables S1.4 and S1.5). It is important to note that the centrosymmetric dimers are translationally aligned approximately along [111] (shown in Fig. 3*c* ▸), with the direction of the π–π interaction at about 15° with respect to [111] towards the *ab* plane, resembling the π–π β-motif (Loots & Barbour, 2012 ▸).

The crystallographic data clearly reveal that the monomer of TAAP belongs to the *C*_1_(1) symmetry group, whereas the dimer is centrosymmetric with the symmetry corresponding to the point group *C_i_* 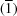, which allows us to categorize the symmetry of its states with two irreducible representations *A_g_* and *A_u_*. According to the exciton theory of Kasha (Gierschner & Park, 2013 ▸; McRae & Kasha, 1958 ▸; Kasha *et al.*, 1965 ▸), the excited state *S*_1_ in H aggregate splits into two energy levels: the lower belonging to the totally symmetric representation *A_g_* and the higher assigned to the representation *A*_*u*_, that is antisymmetric with respect to the inversion. In the ground state, the resultant dipole moment for a centrosymmetric dimer is zero. This is the result of the antiparallel orientation of identical monomer dipole moments. The polarization of the molecules may have an impact not only on the packing motifs in the single-crystal structure but also on the formation of the dimers in solution with the dipole moments of adjacent monomers in an antiparallel configuration.

### 3.2. QTAIM charge-density and NCI analyses   

QTAIM charge-density analysis (Bader, 2003 ▸) was performed for the dimer of TAAP to confirm the existence of π–π interactions in the crystal structure using *AIMAll* software (Keith, 2010 ▸). The wavefunction for calculations was obtained at the DFT/B3LYP 6-311G**(2d,2p) level using *GAUSSIAN09* (Frisch *et al.*, 2009 ▸), which uses experimental geometry with C—H distances fitted to neutron values (Allen & Bruno, 2010 ▸).

**Figure 4:**
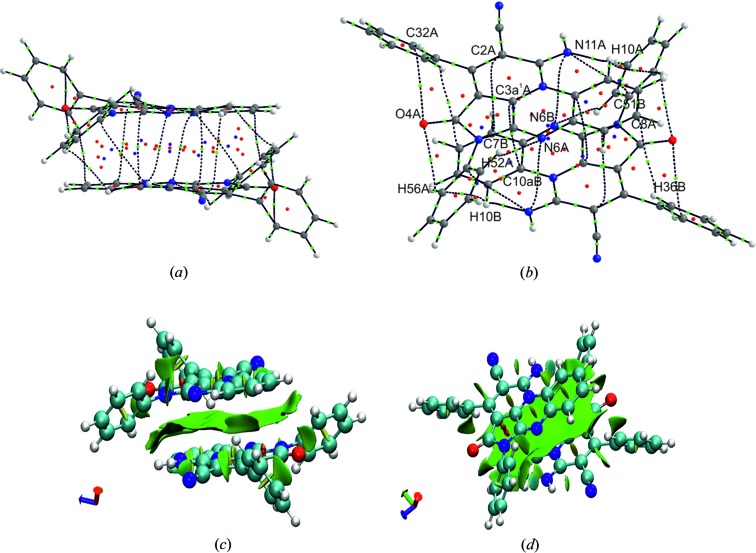
Molecular graph of TAAP with marked critical points: bond critical points (bcp), ring critical points (rcp) and cage critical points (ccp): (*a*) showing a side view and (*b*) a top view. NCI analysis of the intramolecular interactions in the TAAP dimer: (*c*) showing a side view and (*d*) a top view. The blue colour on the plot represents the bonding interaction region, green surfaces represent van der Waals interactions and red surfaces are the non-bonding regions.

Molecular graphs with marked bond critical points (bcp, green), ring critical points (rcp, red), and cage critical points (ccp, blue) are presented in Figs. 4 ▸(*a*) and 4 ▸(*b*). The bcp analysis for the obtained intermolecular interactions is presented in Table S2.1. The existence of three weak intramolecular hydrogen bonds of C—H⋯O or C—H⋯N type (namely H10⋯N11*A*, H56*A*⋯O4*A* and H52*A*⋯N6*A*) was confirmed. These three interactions make the molecular conformation rigid. Additionally, between the two dimer components, there are five pairwise interactions of the N⋯N, C⋯C and N⋯C types, forming π–π interactions and stabilizing the dimer structure. All weak inter- and intramolecular interactions can be classified as closed-shell interactions – |*V*(*r*)|/*G*(*r*) > 1 and ∇^2^ *ρ*(*r*) > 0, *E*(*r*)/*ρ*(*r*) > 0 (Table S6).

*Counterpoise-corrected interaction energy* has been calculated for the dimer of TAAP using the experimental geometry fitted to the neutron *X*—H distances (Allen & Bruno, 2010 ▸) and utilizing the DFT M052X/6–311+G(2df,2p) approach which has proven to be effective for the evaluation of weak interactions (Zhao & Truhlar, 2007 ▸). The basis set superposition error (BSSE) corrected energy is equal to −47.74 kJ mol^−1^. For comparison, the interaction energy using the same method and basis set is −40.08 kJ mol^−1^ in the adenine–tymine stack, −35.44 kJ mol^−1^ in the uracil dimer, and −21.30 kJ mol^−1^ in the water dimer. Aside from the identified π–π interactions, there is also one weak H⋯H bonding interaction (C56*A*—H56*A*⋯H10*B*—C10*B*) and one weak C8*A*⋯H36*B* interaction. NCI analysis was performed to confirm the QTAIM results and to visualize intermolecular interactions between the two dimer components. The NCI method uses the reduced gradient of electron density* s*(*r*) to visualize inter- and intramolecular interactions (Table S2.1). To classify these interactions as favourable or unfavourable, the electron density is multiplied by the sign of second Hessian eigenvalue (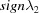). Strong and attractive interactions are those with *ρ*(*r*) > 0 and 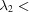 0, whereas for weak interactions *ρ*(*r*) ≃ 0 and 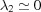 and strong and repulsive interactions are with *ρ*(*r*) > 0 and 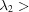 0. Non-covalent interactions can be visualized as isosurfaces, where small red and blue disc-shaped regions represent strong repulsive and attractive interactions, respectively, and broad green and usually irregular surfaces refer to weak interactions. The analysis was performed *via* *NCIPLOT* (Contreras-García *et al.*, 2011 ▸) on the density obtained from the DFT calculations using DFT/B3LYP/6-311G**(2d,2p) with *GAUSSIAN09* (Frisch *et al.*, 2009 ▸) and the results are shown in Figs. 4 ▸(*c*) and 4 ▸(*d*). The NCI analysis confirmed the existence of geometrically predicted inter- and intramolecular interactions responsible for the formation of the stable dimer in the crystal structure. The broad green surface represents the π–π interactions between the π-conjugated heterocyclic rings of TAAP. From the overall shape of the surface, it is evident that dispersion is a major source of attractive forces between the two molecules forming a dimer. This is typical for non-directional π–π interactions and C—H⋯π interactions.

### 3.3. Anisotropy of optical properties   

**Figure 5:**
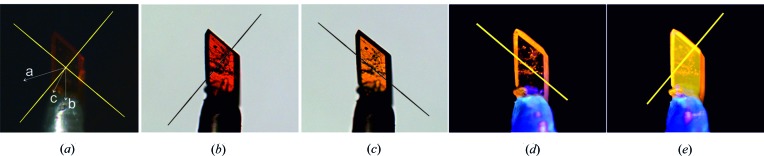
Microscopic images of a TAAP single crystal with the dimensions 0.28 × 0.13 × 0.06 mm obtained for (*a*) crossed polarizers (yellow lines indicate the polarization direction of Nicols); for two different angular orientations of the polarizer producing (*b*) the red colour (*ca* [110]) and (*c*) the yellow colour (*ca* [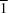
10]) of the crystal, respectively; positions for the crystal illuminated by the light of a UV-LED diode (λ_ex_ = 410 nm) (yellow lines indicate the analyser direction for fluorescence observation) showing (*d*) lack of emission and (*e*) maximum intensity of emission.

The optical properties of biaxial TAAP single crystals were characterized by means of polarized light microscopy. The polarized white-light beam travels perpendicular to the crystallographic (001) face of the crystal. The directions of the plane-polarized light against the crystallographic axes of TAAP are marked in Fig. 5 ▸(*a*). Single crystals of TAAP illuminated with linearly polarized light exhibit different colours depending on the polarization orientation of the incident light, which is known as pleochroism phenomenon (Figs. 5 ▸ *b* and 5 ▸ *c*). Colours ranging from red to yellow are observed as complementary for the absorption of blue–green (at 485–505 nm) and indigo (at 415–445 nm) light, respectively. The approximate orientation of the absorption transition dipole moment (TDM) against the crystal habit and crystallographic axes is along the oscillation direction of the polarized light. The absorption transition dipole moments are located in the orthogonally oriented planes defined by Nicols (compare with Fig. 5 ▸).

We have also observed single-crystal fluorescence anisotropy when the crystal was illuminated by UV–LED diodes with monochromatic nonpolarized light at 410 nm and the emitted fluorescence was examined through a linear polarizing filter (Figs. 5 ▸ *d* and 5 ▸ *e*). Again the light beam travelled perpendicular to the crystallographic (001) face of the crystal. The excitation light with the electric field vector oriented parallel to the transition dipole moment was absorbed. We observed the maximum intensity of emission when the analyzer was oriented along the emission transition dipole moment (Fig. 5 ▸ *e*) that is parallel to the direction of the absorption transition dipole moment (compare with Fig. 5 ▸ *b*).

Relative orientations of the *a*, *b* and* c* crystallographic directions with respect to the crystal habit were determined using the *CrysAlisPro* software on a SuperNova diffractometer (Figs. 5 ▸ *a* and 6 ▸ *a*, and Table S1.1). The fluorescence experiment for the single crystals revealed that the emission transition dipole moment, similarly to that of the absorption transition dipole moment, is oriented in the plane defined by [110] and vector c* perpendicular to the (001) plane.

TAAP in the solid state exhibits a fluorescence emission influenced by restriction of the phenyl-group rotations, formation of intramolecular hydrogen bonds and π-overlapping molecular species (compare Figs. 5 ▸ *e* and 6 ▸ *b*). The fluorescence data for solid TAAP exhibit one band with high intensity at 584 nm. The fluorescence spectrum in the solid state was recorded by excitation at a wavelength of 350 nm, derived from the two-dimensional excitation–emission spectra of TAAP (Fig. S3.1).

### 3.4. Analysis of optical spectra in solution   

The absorption spectra of TAAP were recordered in toluene, CHCl_3_, THF, CH_3_CN and DMSO solutions, as shown in Fig. 7 ▸(*a*). In toluene solution, the absorption maxima appear at 346, 358, 400, 447, 472 and 503 nm with molar absorption coefficients (∊) of 17480, 18130, 6200, 7060, 8200 and 5010 *M* ^−1^cm^−1^, respectively. The maxima at 346, 358, 400, 447, 472 and 503 nm are attributed to π–π^*^ transitions. TAAP exhibits fluorescence emission enhancement in toluene, CHCl_3_, THF, CH_3_CN and DMSO at 527 and 556 nm (Fig. 7 ▸ *b*). The fluorescence quantum yield of TAAP in aceto­nitrile (Φ_i_ = 0.021) was determined using fluorescein as a standard (see Figs. S3.2 and S3.3, and §S4 in the supporting information).

To investigate the possibility of aggregation of TAAP in toluene, CHCl_3_, THF, CH_3_CN and DMSO solutions, the effect of solute concentration on the spectral profiles of the absorption and fluorescence spectra was systematically examined by gradually diluting the respective stock solutions. The absorption spectra for the highest and lowest concentrations were normalized at their respective maxima, unveiling the same spectral characteristic for the bands and no unusual solution behaviour of TAAP in the studied solvents (Figs. S5.1–S5.4). Temperature-dependent optical absorption spectra of TAAP in toluene were recorded at temperatures of 296, 308, 318, 328, 338, 348, 358 and 368 K (Fig. S6.1). It is rather apparent that with temperature increase the aggregate size should decrease. Consequently, at elevated temperatures the relevant absorption and emission spectra should lose their characteristic features. The emission spectra obtained for different concentrations of TAAP in toluene (Figs. S5.5 and S5.6) only reveal the effect of reabsorption, which is especially manifested in the onset region of the fluorescence spectrum for more concentrated samples (see Fig. 7 ▸ *b*). All the fluorescence spectra, corrected for reabsorption and normalized, exhibit a similar pattern (see §S7 in the supporting information). The shift and disappearance of the band may indicate the reabsorption phenomenon (El-Daly & Hirayama, 1997 ▸; Ghosh *et al.*, 2013 ▸) but do not reflect the change of equilibrium between monomer and dimer in solution. Such an equilibrium should be manifested by the changes in the relative intensities of the monomer and dimer features located at wavelengths of 525 nm and 561 nm, respectively. The same effect can be observed in aceto­nitrile, chloro­form and CDCl_3_ or benzene-*d*_6_ (see Figs. S5.5 and S5.6). The influence of the reabsorption on the emission band shapes and shifts is shown in §S7. The corrected fluorescence intensities were calculated for the secondary inner filter effect for TAAP at 100, 50, 10 and 5 µ*M* in aceto­nitrile.

### 3.5. Theoretical calculations of optical properties of TAAP monomers and dimers   

DFT calculations for the optical properties of the TAAP monomer and dimer were performed using *Gaussian09* with B3LYP functional and 6-31G(d,p) basis set (Frisch *et al.*, 2009 ▸) with the aim of revealing the spectral differences between the TAAP monomer and dimer, and to evaluate the possible aggregation type. The calculations were performed in the gas-phase (see §S8 in the supporting information). To simulate spectra in solution, the geometries of both monomer and dimer were fully relaxed, whereas to simulate the solid absorption spectrum, the geometry of the dimer was extracted from the crystal structure. The gas-phase geometry differs from the solid-state geometry only by small changes in the torsion angles of the two phenyl groups: *Ph3* at C30 and *Ph5* at N5. It has also been verified that the solvent effect (from the PCM model) has a marginal effect on the position of the absorption bands. Therefore, the crystal geometry was used in our calculations.

In order to obtain the optical absorption spectra of TAAP down to 250 nm, the lowest 50 singlet states for the monomer and 100 singlet states for the dimer were included. The optical spectra in the gas phase have been computed by smearing the transitions with a Gaussian broadening of 0.15 eV. To obtain the emission spectrum of the dimer, TD-DFT geometry optimization on the *S*_1_ state of the dimer was performed imposing *C_i_* symmetry (see Fig. S8.1).

At the B3LYP level, the calculated spectral bands for the monomer and dimer in the gas phase are blue shifted by ∼0.3 eV with respect to the experimental UV–Vis data of TAAP in toluene and other solvents (Fig. 8 ▸ *a*). This is at odds with the generally good performance of TD-DFT/B3LYP in predicting the absorption spectra of organic molecules (typical blue shift 0.05–0.1 eV). However, it is known that TD-DFT cannot describe properly excitations in certain classes of molecules, characterized either by a multiconfigurational wavefunction or by a large differential correlation between the ground and excited states (Fabian, 2010 ▸). The existence of several resonance structures in TAAP (Fig. 2 ▸) in zwitterionic forms might be an indication of a multiconfigurational character.

In view of these results, a pragmatic approach was adopted and a shift of −0.33 eV to the excitation energies obtained by TD-DFT/B3LYP was applied. This was done in order to align the first excited state of the monomer to the lowest absorption peak of TAAP in solution. The resulting calculated absorption of the monomer is shown in Fig. 8 ▸. From our calculations, we assigned the experimental peak at 472 nm as the first vibronic level of the *S*_0_→*S*_1_ transition (the difference between the 0–0 and 0–1 vibronic levels is ∼1300 cm^−1^, which corresponds to the peak at 1295 cm^−1^ in the IR spectrum). The peak at 448 nm is of electronic origin (*S*_0_→*S*_2_). Note that the calculations reproduce the weak shoulder at 400 nm (*S*_0_→*S*_3_) and the rise of the strong absorption edge at 370 nm and shorter wavelengths.

In Table 1 ▸, the character of the transitions and the orientation of the transition dipole moments (TDM) of the TAAP monomer are reported. In Fig. S8.2, TD-DFT transition densities are shown. To rationalize the electronic transitions, the atoms of TAAP were grouped into three fragments: tri­aza­acephenanthrylene (*tap*), the phenyl ring attached to atom N5 (*Ph5*) and the phenyl ring attached to atom C30 (*Ph3*). Thus, the *S*_0_→*S*_1_ transition is from the imine NH group to *tap*; *S*_0_→*S*_2_ is from *Ph5* to *tap*, *S*_0_→*S*_3_ is from *Ph3* to *tap* and *S*_0_→*S*_4_ is from (*Ph3+Ph5*) to *tap*. All these transitions are of π–π* character and their TDM are parallel to the plane of the molecule. Interestingly, the *S*_0_→*S*_8_ transition is the first one to have a component perpendicular to the plane of the molecule. This weak transition is of π–π* character, and the transition is from *Ph5* to *Ph3*.

To discuss aggregation, the absorption spectrum of the TAAP dimer was calculated and compared to that of the monomer, both in the gas phase. After applying a shift of −0.33 eV, the results are shown in Fig. 8 ▸(*b*). The absorption spectrum of the dimer is red-shifted with respect to the monomer, thus suggesting J-type aggregation. If the interaction between the monomers in the dimer could be approximated by dipole–dipole interaction (Jablonsky diagram, Fig. 1 ▸), each molecular orbital (MO) of the monomer would give rise to a pair of MOs in the dimer, one even (*gerade*) and one odd (*ungerade*), with respect to inversion. As a consequence, each excitation in the dimer would be split into a pair of dark and bright singlets (Davydov splitting) (Basko *et al.*, 2003 ▸; Davydov, 1971 ▸). The bright singlet should have a transition dipole moment twice as large as the corresponding transition in the monomer. In the present case, the π–π interaction between the monomers perturbs this simple picture.

The low lying excitations (*S*_1_ and *S*_2_) come in pairs (dark and bright) and mirror closely in character the first two excited states of the monomer. The TDM of the *S*_0_→*S*_3_ in the dimer at 466 nm (2.66 eV) has a non-negligible component perpendicular to the plane of the *tap*. This transition is similar in character to the *S*_0_→*S*_8_ transition in the monomer at 3.52 eV (352 nm) and shows the largest bathochromic shift from the monomer to the dimer.

### 3.6. Orientation of transition dipole moments   

With the 6-31G(d,p) basis set employed in the TD-DFT calculations, the permanent dipole of the monomer is 3.82 D and the angle α between the dipole vector and the line joining the gravity centres of two TAAP molecules α equals 74.7°. The calculated angle θ between the transition dipole moment and the line joining the centres of gravity, and the calculated angle β between the permanent dipole moment and the transition dipole moment are both given in Table 2 ▸. According to Kasha’s exciton theory (Gierschner & Park, 2013 ▸; McRae & Kasha, 1958 ▸; Kasha *et al.*, 1965 ▸), the nature of the excited states splitting depends on the θ angle. For the aggregate with θ < 54.7°, the electronic transition from the ground to the excited state would lead to the bathochromic shift of the absorption band relative to the monomer, while for aggregates with θ > 54.7°, the shift is hypsochromic.

In the low-lying excited states of the dimer, the angle between the TDM and the line connecting the geometrical centres of the monomers is 54.22°, which is very close to the critical angle of 54.7°. The value of θ angle suggests that the aggregate and the isolated monomer should have absorption bands at almost the same wavelength. In this case, the determination of the aggregation type on the basis of absorption spectra is inconclusive.

To explain why H-type aggregation causes bathochromical shift, the molecular orbital energy levels of the dimer were analysed (see Fig. S8.1). The low-lying excitations are dominated by one single transition, with only a minor admixture from other energy levels. We have found that the splitting of dimer HOMO and HOMO–1 is large (0.08 eV), while that of LUMO and LUMO+1 is negligible. The HOMO and HOMO–1 levels of the dimer are raised in energy with respect to monomer HOMO. As a result, the symmetry-permitted transitions of the dimer are lower in energy with respect to the HOMO–LUMO transition of the monomer. Since the lowest singlet excitation is not allowed by symmetry, this suggests H-type aggregation, even though the overall absorption spectrum is bathochromically shifted, as for the J-type. This could suggest that electrostatic interactions between monomers with permanent dipole moments (*ca* 4 D) each lead to the unusual spectroscopic JH-aggregate behaviour of the TAAP dimer.

### 3.7. ^1^H NMR data for TAAP in solution   

To substantiate the formation of either monomer or dimer in solution, NMR experiments were performed. The ^1^H NMR spectra of TAAP were measured in CDCl_3_, CD_2_Cl_2_, benzene-*d*_6_, toluene-*d*_8_, THF-*d*_8_ and dioxane-*d*_8_, and display only one set of signals (Fig. 9 ▸ *a*). The ^1^H NMR spectra of TAAP, detected at low and high concentrations in CDCl_3_ and benzene-*d*_6_, do not show any changes of chemical shifts and broadening of the signals (§S9, Figs. S9.1–S9.4). Temperature-dependent spectra in tetra­chloro­ethane measured in the range 296–345 K indicated no equilibrium between the monomer and dimer. In addition, the ^1^H diffusion-ordered spectroscopy (DOSY) NMR (Avram & Frish, 2005 ▸) spectrum shows only one set of signals at diffusion coefficient log*D* = −8.38, indicating the presence of only one form in CDCl_3_ (Fig. 9 ▸ *b*). The attempt to identify this form using nuclear Overhauser effect (NOE) spectroscopy was undertaken. Full assignment of the H and C atoms of TAAP was performed by two-dimensional COSY, HSQC and HMBC measurements in THF-*d*_8_ (see §S10). The through-space correlations were determined by ROESY spectra only with the monomer measured in THF-*d*_8_. No intermolecular cross coupling between H atoms of the two monomers in the dimer have been found. We obtained the same results performing the two-dimensional NMR measurements in dioxane-*d*_8_.

The only evidence of two different species was found in the DOSY spectra of TAAP at 345 K in tetra­chloro­ethane-*d*_2_ [log*D* = −9.32 and log*D* = −9.28, Fig. 9( ▸ *c*)]. The set of signals at log*D* = −9.32 contained features that are characteristic of spin systems observed in one-dimensional ^1^H NMR spectra, detected in different solvents (Fig. 9 ▸ *a*), whereas the set at log*D* = −9.28 indicated the downfield shift of the H-10 signal from 10.32 ppm to the aromatic region. The small difference between the log*D* values may indicate a similar hydro­dynamic radius for both species.

In order to determine the equilibrium between the monomer and dimer species, a measurement of the permanent dipole moment of TAAP in saturated 1,4-dioxane solution was performed (see §S11 in the supporting information). The experimentally obtained value of 4.24 D at 300 K is in good agreement with the calculated ground-state dipole moment [3.82 D by TD-DFT/6-31G(d,p) basis set and 4.94 D by DFT/B3LYP/6-311G^**^(2d,2p), Fig. S2.1] and indicates that the equilibrium is shifted almost exclusively to the monomer form.

Single crystals of TAAP were obtained from a cooled tetra­chloro­ethane-*d*_2_ solution in an NMR cuvette. X-ray diffraction experiments confirmed that the crystal structure was in fact the same *P* 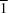 form found for TAAP crystallized from all other solvents.

### 3.8. Spectroscopic properties of single crystals of TAAP   

**Figure 6:**
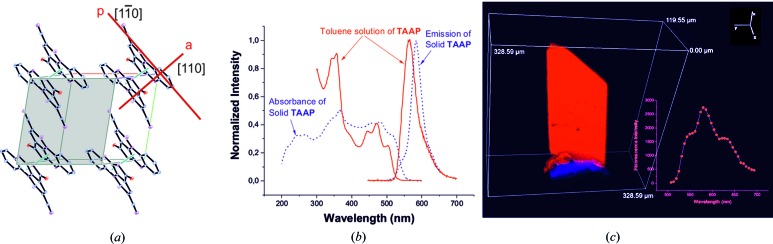
(*a*) View of the TAAP crystal structure at RT projected onto the (001) plane, parallel to the largest face of the crystal used for the fluorescence experiment (H atoms and phenyl rings at C3 and C5 have been omitted for clarity and displacement ellipsoids are drawn at the 30% probability level). The maximum fluorescence was observed with the analyzer approximately along [110]; the distances between gravity centres of molecules in the dimer (3.495 Å for TAAP_RT and 3.417 Å for TAAP_LT) are shown as turquoise lines. The orientation of the polarizers (compare with Fig. 5 ▸), approximately along [1
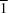
0] and [110], are marked by red lines. (*b*) Normalized absorption and fluorescence spectra for the solution of TAAP in toluene (solid line) and for the TAAP polycrystalline sample (double-dashed line). (*c*) The fluorescence three-dimensional image of the TAAP single crystal (λ_ex_ = 405 nm). Inset: fluorescence spectrum for a TAAP single crystal (λ_ex_ = 405 nm).

**Figure 7:**
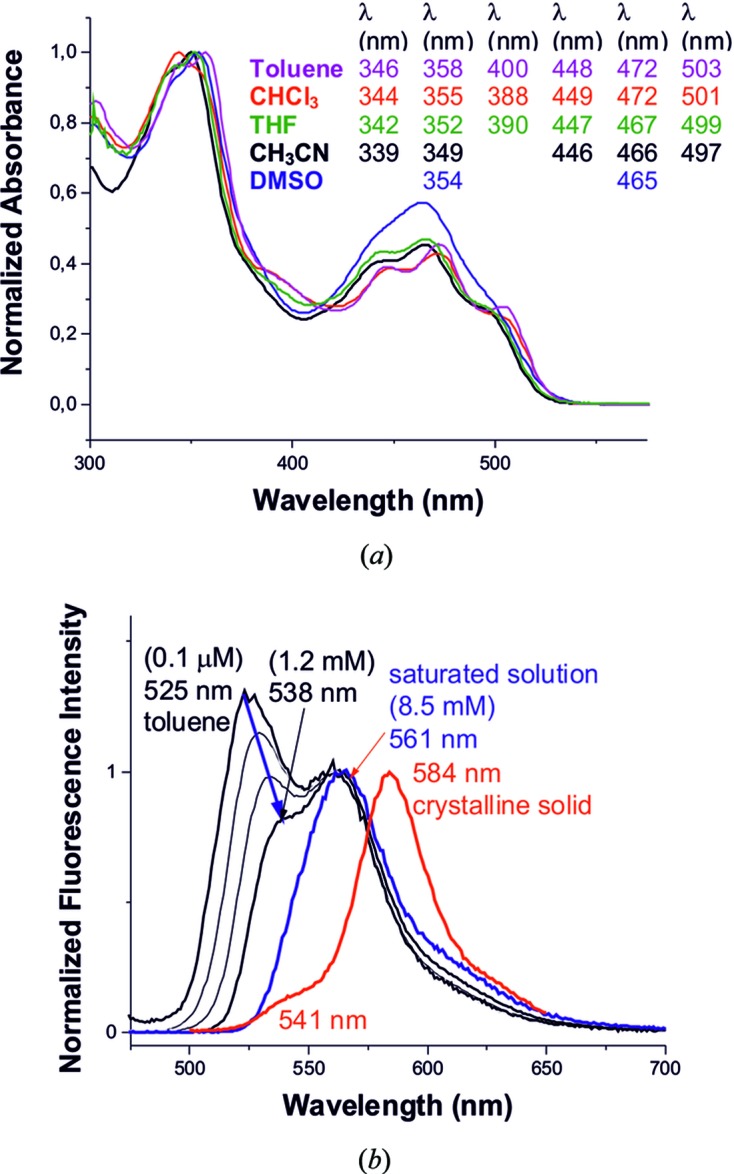
(*a*) Normalized absorption spectra of TAAP in toluene, CHCl_3_, THF, CH_3_CN and DMSO. (*b)* Normalized fluorescence spectra (to the height of the band at 561 nm) at various concentrations of TAAP in toluene (λ_ex_ = 440 nm) and fluorescence spectrum of TAAP in the crystalline state (red line, λ_ex_ = 350 nm).

**Figure 8:**
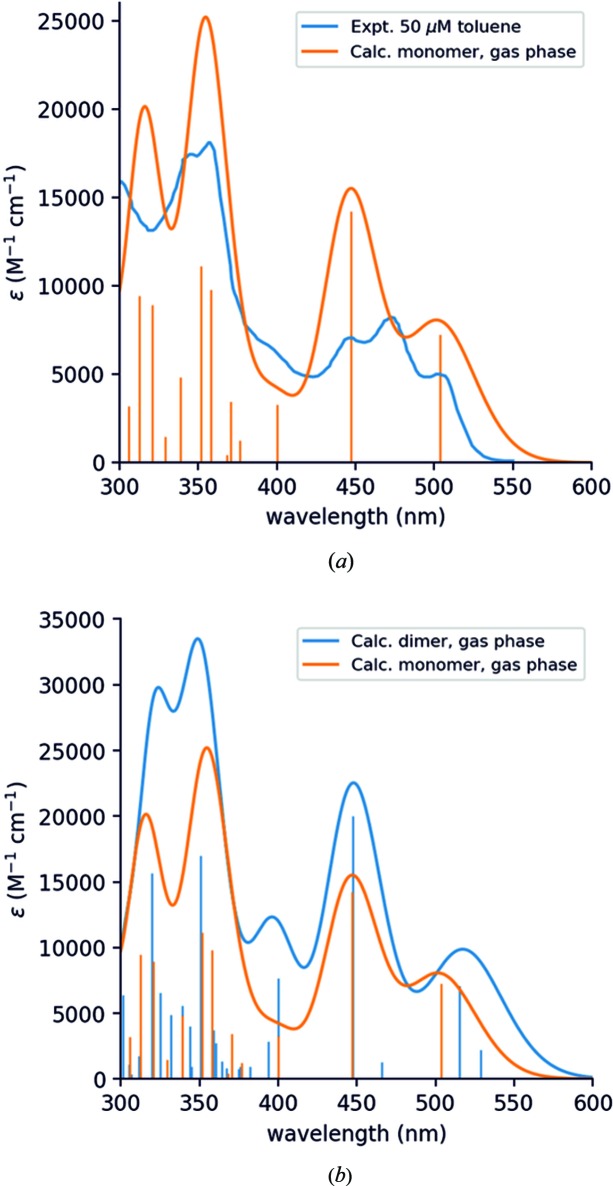
(*a*) Absorption spectra of the TAAP monomer in the gas phase, computed at the TD-DFT/B3LYP level (shifted by −0.33 eV), compared to the experimental absorption spectra of TAAP in toluene (50 µ*M*). (*b*) Absorption spectra of the TAAP monomer and dimer in the gas phase, computed at the TD-DFT/B3LYP level (shifted by −0.33 eV).

**Figure 9:**
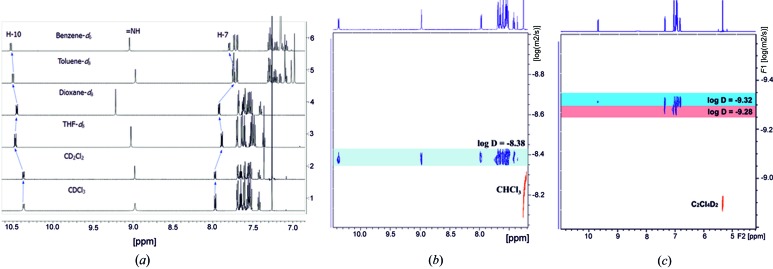
(*a*) ^1^H NMR spectra of TAAP in CDCl_3_, CD_2_Cl_2_, THF-*d*_8_, dioxane-*d*_8_, toluene-*d*_8_ and benzene-*d*_6_. (*b*) ^1^H DOSY NMR spectrum of TAAP in CDCl_3_. (*c*) ^1^H DOSY NMR spectrum of TAAP in tetra­chloro­ethane-*d*_2_ at 345 K.

The photophysical properties of TAAP single crystals were investigated using UV–vis absorption and photoluminescence spectroscopy. UV–vis spectra were measured in order to determine the aggregation type on the basis of the band spectral shifts. The absorption spectra of TAAP crystals revealed three bathochromically shifted band maxima at 456, 482 and 509 nm (Figs. 6 ▸ and 7 ▸). The formation of the dimer by molecules with permanent dipole moments is manifested strongly in the 55 nm red-shift of the UV–vis absorption bands, attributed to the *S*_0_→*S*_1_ transition with respect to those recorded for TAAP in solution.

**Figure 10:**
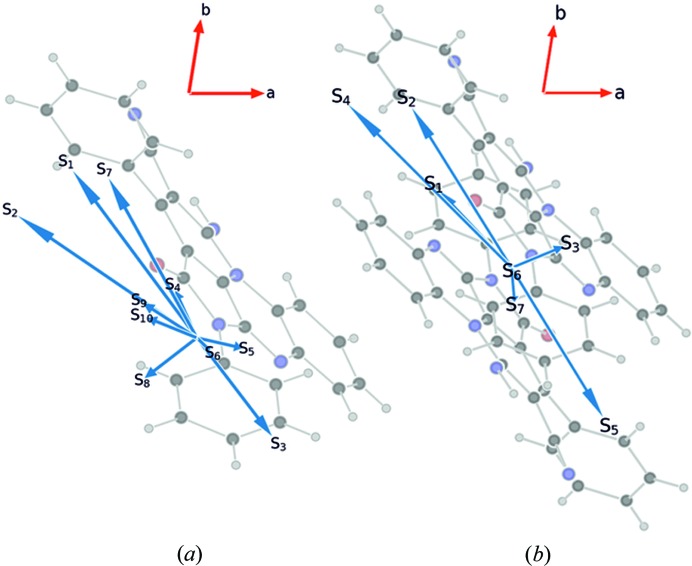
The orientation of the calculated transition dipole moments (TDMs) for (*a*) the TAAP monomer and (*b*) the TAAP dimer in relation to both the chromophore plane and the crystallographic plane (001). All TDMs except *S*_8_ of the monomer and *S*_3_ of the dimer are approximately aligned along the [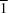
10] direction.

In order to explain the anisotropy of the optical properties, we analyzed the TDM of the monomer and the dimer, reporting them in the reference frame of the crystal, with the (001) plane parallel to plane of the paper. The calculated TDMs of the low-lying excitations are shown in Fig. 10 ▸. In the monomer, all but the *S*_0_→*S*_8_ transition have a TDM component approximately parallel to the [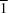
10] direction. The intermolecular interactions cause red-shifted *S*_0_→*S*_8_ transitions, which becomes the *S*_0_→*S*_3_ transition in the dimer and has a large component along the [110] direction. The closest crystallographic directions (with low Miller indices) are reported in the last column of Tables 1 ▸ and 2 ▸.

**Figure 11:**
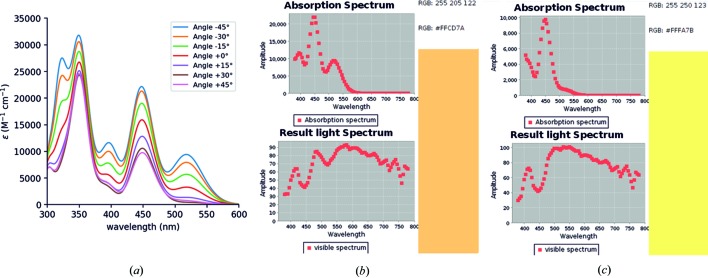
(*a*) Calculated absorption spectra of the dimer as a function of the photon polarization axis. (*b*) Perceived colour for the polarization axis at −45° against the *b*-crystallographic direction in the (001) plane (orange–red). (*c*) Perceived colour for the polarization axis at +45° (yellow). The crystallographic and polarizer directions are the same as in Fig. 5 ▸.

To simulate the effect of polarized light, the absorption spectrum of the dimer was computed, by setting the dipole component perpendicular to the polarization plane of the light to zero. The result is shown in Fig. 11 ▸. The perceived RGB colour of TAAP was determined by subtracting the absorption spectrum from the spectrum of visible light (Broadbent, 2004 ▸) as a function of the polarization direction. When the polarizer is rotated at −45° (roughly in the [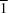
10] direction), TAAP absorbs both in the blue and green regions, hence the perceived colour is orange–red. Conversely, when the polarized light is rotated at +45° (roughly in the [110] direction), TAAP absorbs mostly in the indigo–blue region, thus the perceived colour is yellow.

Finally, the spectra of solid TAAP were simulated from the absorption and emission spectra of the dimer, taking into account the effect of the solid environment, allowing for the influence of the electric field generated by the neighbouring molecules within the Clausius–Mossotti (CM) approximation (see §S12 in the supporting information):
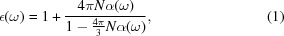
where *ε(ω)* is the complex dielectric function, *N* is the number density of dimers in the crystal and *α(ω)* is the molecular polarizability calculated from TD-DFT. The CM approximation is valid in principle only for an isotropic and uniform medium and is equivalent to space-averaging the local field effects. Then, we calculated the absorbance η from the extinction coefficient κ: 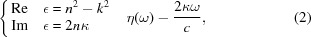
where *n* is the refraction index, *ω* is the frequency and *c* is the speed of light.

**Figure 12:**
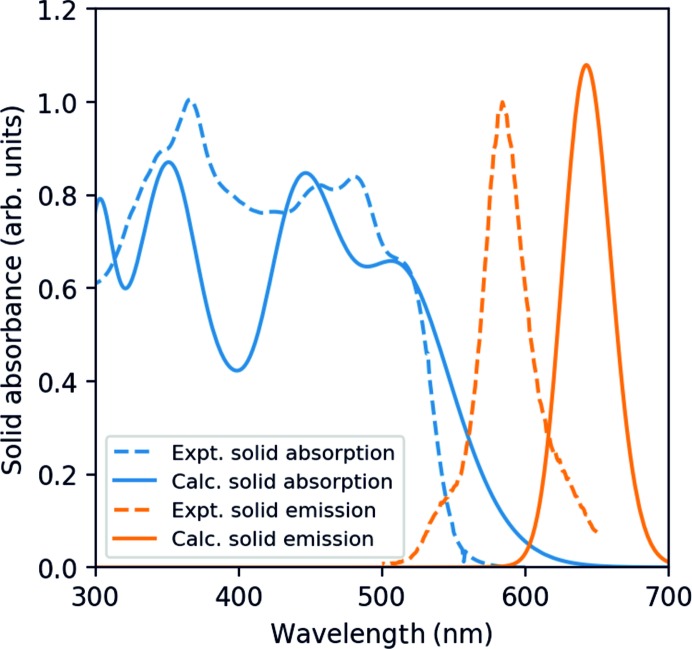
Comparison of the calculated spectra of the TAAP crystal in the Clausius–Mossotti approximation with the experimental spectra.

The result is shown in Fig. 12 ▸ and compared to the experimental spectra. The calculated absorption is in fair agreement with the experimental spectrum. It is clear that in the crystal phase the small splitting of the LUMO in the dimer may lead to the additional electronic coupling in the Frenkel exciton states and the resultant broadening of states.

The emission peak was calculated as the vertical energy difference between the *S*_1_ and *S*_0_ states, both in the geometry of *S*_1_ relaxed by TD-DFT/B3LYP. The calculated emission has been rescaled to match the normalized experimental spectrum and is slightly red-shifted (645 nm, 1.92 eV) with respect to the experiments. The main differences between the *S*_0_ and *S*_1_ geometry are the dihedral angles of the two phenyl groups. The *Ph5* torsion changes from 44.3 to 22.7° in the excited state (experimental value 41.37°), while the *Ph3* torsion changes from 55.1 to 43.7° in the excited state (experimental value 58.28°). As a matter of fact, the Frenkel exciton can be delocalized on several dimers and the crystalline packing will reduce the change in the torsion angles. Consequently, the emission energy is expected to increase, which is in better agreement with the experiment.

## 4. Conclusions   

We have demonstrated that optically biaxial single crystals of 5,6,10b-tri­aza­acephenanthrylene (TAAP) exhibit absorption and fluorescence anisotropy upon JH-aggregation with a face-to-face alignment of the monomers in centrosymmetric dimers. Sole analysis of the optical spectra of TAAP in solution and the solid state does not allow us to define the aggregation type unambiguously. DFT calculations for the TAAP dimer in the gas phase indicate that the lowest singlet excitation is forbidden by symmetry, suggesting H-type aggregation, even though the overall absorpton spectrum is bathochromically shifted, as for the J-type. The calculated value of θ was found to be 54.22°, which is very close to the critical angle of 54.7°, showing an unusual example of the JH-aggregation type with nearly the same absorption bands for both monomer and dimer. The experimental determination of the permament dipole moment of the TAAP molecule in 1,4-dioxane solution indicates unambiguously that the monomer form is predominantly present in solution.

The π–π interaction between monomer units in the dimer, with an interplanar distance of 3.413 Å between the chromophores and an interaction energy of −11.41 kcal mol^−1^, are responsible for both the crystal packing arrangement and relative orientations of the absorption and emission transition dipole moments. The absorption and emission spectra calculated for a crystal of TAAP in the Clausius–Mossotti approximation sustain the experimentally determined orientation of the absorption and emission transition dipole moments in the TAAP single crystal.

The π–π interactions are usually one of the major reasons for reduced or even absent fluorescence of materials in aggregates or the solid state. Our findings will aid the understanding of the nature of absorption and fluorescence emission anisotropy, observed for the crystalline JH-aggregates with potential applications as ambipolar transporting organic materials (Park *et al.*, 2013 ▸).

## Supplementary Material

Crystal structure: contains datablock(s) 3358_2a_RT, 3157_2a_LT. DOI: 10.1107/S2052252518001987/yc5013sup1.cif

Structure factors: contains datablock(s) shelx. DOI: 10.1107/S2052252518001987/yc50133358_2a_RTsup2.hkl

Structure factors: contains datablock(s) shelx. DOI: 10.1107/S2052252518001987/yc50133157_2a_LTsup3.hkl

Supporting information. DOI: 10.1107/S2052252518001987/yc5013sup4.pdf

CCDC references: 1821491, 1821492

## Figures and Tables

**Table 1:** Low-lying optical transitions (singlets only) of the TAAP monomer in the gas phase, calculated at the TD-DFT/B3LYP level. The transition wavelengths (λ_s_) are obtained after shifting the TD-DFT transition energies by −0.33 eV (∊_s_). H is the highest occupied molecular orbital (HOMO) and L is the lowest unoccupied molecular orbital (LUMO). β is the angle between the direction of the permanent dipole moment (PDM) and the transition dipole moment (TDM)

Ex. state	∊_s_ (eV)	λ_s_ (nm)	Oscillator strength	Dominant trans. (coeff)	Character	TDM (Debye)	β (°)	γ (°)	Best cryst. direction
*S*_1_	2.46	503	0.0726	H→L (0.64)	(*tap* + NH)→tap*	2.62	19.43	4.35	[ 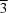 40]
*S*_2_	2.77	447	0.1424	H-1→L (0.63)	*Ph5*→tap*	3.48	59.92	0.34	[ 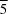 34]
*S*_3_	3.10	400	0.0329	H-2→L (0.66)	*Ph3*→tap*	1.59	30.23	6.90	[3 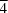 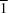 ]
*S*_4_	3.29	377	0.0160	H-3→L (0.61)	*Ph3*+*Ph5*→tap*	0.96	30.23	2.54	[2 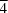 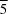 ]
…	…	…	…	…	…	…	…	…	...
*S*_8_	3.52	352	0.1114	H-6→L (0.20), H-1→L+1 (0.43), H→L+1 (0.40)	*Ph5*+*tap*→(*Ph3*+*tap*)*	2.78	71.95	0.68	[ 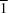 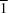 5]

**Table 2:** Low-lying optical transitions (singlets only) of the TAAP dimer in the gas phase, calculated at the TD-DFT/B3LYP level. The transition wavelengths (λ_s_) are obtained after shifting the TD-DFT transition energies by −0.33 eV (∊_s_). H is the HOMO and L is the LUMO. β is the angle between the direction of the permanent dipole moment (PDM) and the transition dipole moment (TDM). γ is the complementary angle between the TDM and the normal to the plane of the molecule. θ is the angle between the TDM and the vector connecting the centre of mass of the two molecules in the dimer, n.d. is not defined

Ex. state	∊_s_ (eV)	λ_s_ (nm)	Oscillator strength	Dominant trans. (coeff)	Character	TDM (Debye)	β (°)	θ (°)	γ (°)	Ex. state
*S*_1_ dark	2.32	553	0	H→L+1 (0.67)	*tap*→tap*	0	n.d.	n.d.	n.d.	n.d.
*S*_1_ bright	2.34	529	0.0225	H→L (0.62)	*tap*→tap*	1.49	34.08	54.22	2.63	[ 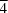 41]
*S*_2_ dark	2.40	517	0	H-1→L (0.66)	*Ph5*+*tap*→tap*	0	n.d.	n.d.	n.d.	n.d.
*S*_2_ bright	2.41	515	0.0709	H-1→L+1 (0.61)	*Ph5*+*tap*→tap*	2.61	12.17	67.62	5.98	[ 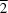 30]
*S*_3_ dark	2.65	468	0	H-2→L+1 (0.68)	*Ph5*→tap*	0	n.d.	n.d.	n.d.	n.d.
*S*_3_ bright	2.66	466	0.0131	H-2→L (0.66)	*Ph5*→tap*	1.07	86.51	72.84	25.38	[31 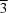 ]
*S*_4_ dark	2.71	457	0	H-3→L (0.68)	*Ph5*→tap*	0	n.d.	n.d.	n.d.	n.d.
*S*_4_ bright	2.77	447	0.2000	H-3→L+1 (0.63)	*Ph5*→tap*	4.12	58.9	32.29	8.46	[ 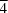 45]
