# Supplementary material for: π–π-Induced aggregation and single-crystal fluorescence anisotropy of 5,6,10b-tri­aza­acephenanthrylene
Source: IUCrJ. 2018 Apr 18;5(Pt 3):335–47. doi: 10.1107/S2052252518001987 (PMC5929379; doi:10.1107/S2052252518001987)
Supplement: Supplementary file 4 [file m-05-00335-sup4.pdf]

IUCrJ

Volume 5 (2018)

Supporting information for article:

π-π Induced Aggregation and Single Crystal Fluorescence Anisotropy of 5,6,10b-Triazaacephenanthrylene

Katarzyna Ostrowska, Davide Ceresoli, Katarzyna Stadnicka, Marlena Gryl, Marco Cazzaniga, Raffaella Soave, Bogdan Musielak, Łukasz J. Witek, Piotr Goszczycki, Jarosław Grolik and Andrzej M. Turek

Supporting Information

Crystallographic data for TAAP_LT and TAAP_RT (S1), Non-Covalent Interactions analysis (S2), 2D fluorescence spectra of TAAP in crystalline solid and solution (S3), determination of fluorescence quantum yield of TAAP in CH_3CN (S4), fluorescence concentration-dependent spectra of TAAP in CH_3CN and $CHCl_3$ (S5), temperature-dependent fluorescence spectra of TAAP at temperature range 296 - 361 K (S6), correction of fluorescence intensity for the inner filter effects for TAAP (5 µM, 10 µM, 50 µM, and 100 µM in acetonitrile) (S7), DFT calculation (S8), 1D fluorescence spectra of TAAP and ^{1}H NMR data of TAAP measured at the same concentrations 0.24 mM and 0.1 M in benzene-d_6 (S9), 2D NMR data for TAAP in THF-d_8 (S10), determination of experimental permanent dipole moment (S11), details of Clausius-Mossotti model for optical absorption of molecular solids (S12).

S1. Crystallographic data for TAAP_LT and TAAP_RT.

Table S1.1.

Crystal data, intensity measurement conditions and structure refinement details for two experiments performed TAAP at T=130(1) K (TAAP_LT) and T=293(1) K (TAAP_RT). The single crystal TAAP_RT was also used for the latter fluorescence experiment.

**Identification code**	**TAAP_LT**	**TAAP_RT**
Crystal data		
Chemical formula	$C_{26}H_{15}N_5O$	$C_{26}H_{15}N_5O$
Mr	413.43	413.43
Wavelength (Å)	0.71073	1.54184
Temperature (K)	130 (1)	293 (1)
Crystal system	Triclinic	Triclinic
Space group	$P\bar{1}$	$P\bar{1}$
Unit cell dimensions (Å, º)	*a* = 9.2591 (4)	*a* = 9.3048 (4)
	b = 9.4186 (3)	*b* = 9.5725 (4)
	c = 11.9361 (5)	*c* = 12.0202 (7)
	α = 83.980 (3)	α = 82.616 (4)
	β = 67.267 (4)	β = 67.249 (5)
	γ = 80.944 (3)	γ = 79.675 (4)
V (Å^3)	946.98(7)	969.28 (9)
Z, Dx (Mg/m^3)**	2, 1.450	2, 1.417
μ (mm^{-1})	0.093	0.726
F (000)	428	428
Crystal size (mm)	0.42x0.30x0.10	0.28x0.13x0.06
Data collection		
θ Range (º)	2.938 – 31.881	3.996 - 69.996
Method	ω scans	ω scans
Reflections collected	29028	14738
Reflections unique	6185	3685
R(int)	0.0488	0.0314
Reflections $I > 2\sigma(I)$	4465	2568
Completeness (θ full)	0.999	1.000
T_{min}, T_{max}	0.962, 0.991	0.823, 0.958
Refinement		
Data/restraints/parameters	6185 / 0 / 292	3685 / 1 /292
Goodness-of-fit	1.045	1.032
$R1$ [$I > 2\sigma(I)$]	0.0505	0.0463
$wR2$ (all data)	0.1446	0.1377
Weighting scheme: A, B	0.0681, 0.2810	0.0659, 0.2026
$\Delta\rho_{max}$ $\Delta\rho_{min}$ rms (e Å^{-3})	0.413 -0.290 0.058	0.226 -0.169, 0.036

Diffractometer: Agilent SuperNova, Computer programs: CrysAlisPro [CrysAlisPro, Oxford Diffraction Ltd., Version 1.171.33.66], SIR92 [Altomare, A., Cascarano, G., Giacovazzo, C., Guagliardi, A., Burla, M. C., Polidori, G. & Camalli, M. (1994). *J. Appl. Crystallogr.* 27, 435], SHELXL - CRYSTAL STRUCTURE REFINEMENT - MULTI-CPU VERSION [Copyright(C) George M. Sheldrick 1993-2013, Version 2013/4, G.M. Sheldrick, *Acta Crystallogr*. C71 (2015) 3-8]; $w = 1/[\sigma^2(F_o^2) + AP^2 + BP]$, where $P = (F_o^2+2F_c^2)/3$.

Table S1.2.

Selected bond lengths (Å), valence, torsion and dihedral angles (º) for TAAP_LT at T=130(1) K

**Bond lengths**	
C(1)-N(11)	1.269 (2)
C(1)-N(10B)	1.444 (2)
C(1)-C(2)	1.480 (2)
C(2)-C(3)	1.379 (2)
C(2)-C(21)	1.437 (2)
C(3)-C(3A)	1.420 (2)
C(3)-C(31)	1.480 (2)
C(3A)-C(3A')	1.367 (2)
C(3A)-C(4)	1.474 (2)
C(4)-O(4)	1.214 (2)
C(4)-N(5)	1.426 (2)
N(5)-C(5A)	1.391 (2)
N(5)-C(51)	1.432 (2)
C(5A)-N(6)	1.289 (2)
C(5A)-C(3A')	1.445 (2)
N(6)-C(6A)	1.401 (2)
C(6A)-C(7)	1.406 (2)
C(6A)-C(10A)	1.424 (2)
C(7)-C(8)	1.380 (2)
C(8)-C(9)	1.394 (2)
C(9)-C(10)	1.390 (2)
C(10)-C(10A)	1.399 (2)
C(10A)-N(10B)	1.430 (2)
N(10B)-C(3A')	1.350 (2)
C(21)-N(22)	1.149 (2)
C(31)-C(32)	1.396 (2)
C(31)-C(36)	1.401 (2)
C(32)-C(33)	1.388 (2)
C(33)-C(34)	1.383 (2)
C(34)-C35)	1.389 (2)
C(35)-C(36)	1.388 (2)
C(51)-C(52)	1.386 (2)
C(51)-C(56)	1.393 (2)
C(52)-C(53)	1.393 (2)
C(53)-C(54)	1.389 (2)
C(54)-C(55)	1.384 (2)
C(55)-C(56)	1.390 (2)
**Valence angles**	
N(11)-C(1)-N(10B)	119.1 (1)
N(11)-C(1)-C(2)	126.6 (1)
N(10B)-C(1)-C(2)	114.4 (1)
C(3)-C(2)-C(21)	120.6 (1)
C(3)-C(2)-C(1)	126.0 (1)
C(21)-C(2)-C(1)	113.4 (1)
C(2)-C(3)-C(3A)	115.0 (1)
C(2)-C(3)-C(31)	122.1 (1)
C(3A)-C(3)-C(31)	122.8 (1)
C(3A')-C(3A)-C(3)	119.3 (1)
C(3A')-C(3A)-C(4)	106.9 (1)
C(3)-C(3A)-C(4)	133.8 (1)
O(4)-C(4)-N(5)	123.9 (1)
O(4)-C(4)-C(3A)	129.9 (1)
N(5)-C(4)-C(3A)	106.1 (1)
C(5A)-N(5)-C(4)	110.0 (1)
C(5A)-N(5)-C(51)	126.0 (1)
C(4)-N(5)-C(51)	123.9 (1)

N(6)-C(5A)-N(5)	128.9 (1)
N(6)-C(5A)-C(3A')	124.9 (1)
N(5)-C(5A)-C(3A')	106.2 (1)
C(5A)-N(6)-C(6A)	114.3 (1)
C(7)-C(6A)-N(6)	116.3 (1)
C(7)-C(6A)-C(10A)	118.6 (1)
N(6)-C(6A)-C(10A)	125.1 (1)
C(8)-C(7)-C(6A)	121.3 (1)
C(8)-C(7)-C(9)	119.5 (1)
C(10)-C(9)-C(8)	120.9 (1)
C(9)-C(10)-C(10A)	120.0 (1)
C(10)-C(10A)-C(6A)	119.6 (1)
C(10)-C(10A)-N(10B)	123.2 (1)
C(6A)-C(10A)-N(10B)	117.2 (1)
C(3A')-N(10B)-C(10A)	116.9 (1)
C(3A')-N(10B)-C(1)	116.7 (1)
C(10A)-N(10B)-C(1)	126.4 (1)
N(10B)-C(3A')-C(3A)	128.0 (1)
N(10B)-C(3A')-C(5A)	121.3 (1)
C(3A)-C(3A')-C(5A)	110.7 (1)
N(22)-C(21)-C(2)	176.5 (2)
C(32)-C(31)-C(36)	119.6 (1)
C(52)-C(51)-C(56)	120.7 (1)
**Torsion angles**	
C(2)C(3)C(31)C(32)	-121.8 (2)
C(2)C(3)C(31)C(36)	54.8 (2)
C(3A)C(3)C(31)C(32)	54.2 (2)
C(3A)C(3)C(31)C(36)	-129.2 (2)
C(4)N(5)C(51)C(52)	-138.8 (1)
C(4)N(5)C(51)C(56)	40.8 (20
C(5A)N(5)C(51)C(52)	44.5 (2)
C(5A)N(5)C(51)C(56)	-136.0(1)
**Dihedral angles**	
Best Plane 1/ Best Plane 2	0.62 (2)
Best Plane 3/ Best Plane 2	0.75 (4)
Best Plane 4/ Best Plane 2	58.28 (4)
Best Plane 5/ Best Plane 2	41.37 (5)
Best Plane 6/ Best Plane 2	4.95 (5)
Best Plane 6/ Best Plane 3	5.42 (6)

Best Plane 1 :
C(1)C(2)C(3)C(3A)C(3A')C(4)C(5)C(5A)N(6)C(6A)C(7)C(8)C(9)C(10)C(10A)N(10B)N(11)C(21)N(21) [19 atoms]
Best Plane 2 : C(1)C(2)C(3)C(3A)C(3A')C(4)C(5)C(5A)N(6)C(6A)C(7)C(8)C(9)C(10)C(10A)N(10B) [16 atoms]
Best Plane 3 : C(3A)C(4)C(5)C(5A)N(6)C(6A)C(7)C(8)C(9)C(10)C(10A)N(10B)C(3A') [13 atoms]
Best Plane 4 : C(31)C(32)C(33)C(34)C(35')C(36)
Best Plane 5 : C(51)C(52)C(53)C(54)C(55)C(56)
Best Plane 6 : C(1)C(2)C(3)C(3A)C(3A')N(10B)

Table S1.3.

Selected bond lengths (Å), valence, torsion and dihedral angles (º) for TAAP_RT at T=293(1) K

Bond lengths

C(1)-N(11)	1.241 (3)
C(1)-N(10B)	1.443 (3)
C(1)-C(2)	1.473 (3)
C(2)-C(3)	1.377 (3)
C(2)-C(21)	1.435 (3)
C(3)-C(3A)	1.416 (3)
C(3)-C(31)	1.479 (3)
C(3A)-C(3A')	1.363 (3)

C(3A)-C(4)	1.469 (3)
C(4)-O(4)	1.207 (2)
C(4)-N(5)	1.427 (2)
N(5)-C(5A)	1.387 (2)
N(5)-C(51)	1.435 (2)
C(5A)-N(6)	1.280 (2)
C(5A)-C(3A')	1.436 (3)
N(6)-C(6A)	1.416 (3)
C(6A)-C(7)	1.388 (3)
C(6A)-C(10A)	1.414 (3)
C(7)-C(8)	1.369 (3)
C(8)-C(9)	1.379 (3)
C(9)-C(10)	1.377 (3)
C(10)-C(10A)	1.393 (3)
C(10A)-N(10B)	1.429 (2)
N(10B)-C(3A')	1.350 (2)
C(21)-N(22)	1.141 (3)
C(31)-C(32)	1.391 (3)
C(31)-C(36)	1.386 (3)
C(32)-C(33)	1.379 (3)
C(33)-C(34)	1.360 (4)
C(34)-C35)	1.383 (4)
C(35)-C(36)	1.392 (3)
C(51)-C(52)	1.370 (3)
C(51)-C(56)	1.385 (3)
C(52)-C(53)	1.387 (3)
C(53)-C(54)	1.360 (4)
C(54)-C(55)	1.378 (3)
C(55)-C(56)	1.377 (3)

Valence angles

N(11)-C(1)-N(10B)	119.9 (2)
N(11)-C(1)-C(2)	125.9 (2)
N(10B)-C(1)-C(2)	114.2 (2)
C(3)-C(2)-C(21)	120.7 (2)
C(3)-C(2)-C(1)	126.2 (2)
C(21)-C(2)-C(1)	113.1 (2)
C(2)-C(3)-C(3A)	115.0 (2)
C(2)-C(3)-C(31)	122.2 (2)
C(3A)-C(3)-C(31)	122.8 (2)
C(3A')-C(3A)-C(3)	119.5 (2)
C(3A')-C(3A)-C(4)	106.8 (2)
C(3)-C(3A)-C(4)	133.7 (2)
O(4)-C(4)-N(5)	123.8 (2)
O(4)-C(4)-C(3A)	130.3 (2)
N(5)-C(4)-C(3A)	105.9 (2)
C(5A)-N(5)-C(4)	110.1 (2)
C(5A)-N(5)-C(51)	126.0 (2)
C(4)-N(5)-C(51)	123.8 (2)
N(6)-C(5A)-N(5)	128.9 (2)
N(6)-C(5A)-C(3A')	125.2 (2)
N(5)-C(5A)-C(3A')	105.9 (2)
C(5A)-N(6)-C(6A)	114.4 (2)
C(7)-C(6A)-N(6)	116.5 (2)
C(7)-C(6A)-C(10A)	119.0 (2)
N(6)-C(6A)-C(10A)	124.5 (2)
C(8)-C(7)-C(6A)	121.2 (2)
C(9)-C(8)-C(7)	119.7 (2)
C(10)-C(9)-C(8)	120.9 (2)
C(9)-C(10)-C(10A)	120.1 (2)
C(10)-C(10A)-C(6A)	119.1 (2)

C(10)-C(10A)-N(10B)	123.4 (2)
C(6A)-C(10A)-N(10B)	117.4 (2)
C(3A')-N(10B)-C(10A)	117.1 (2)
C(3A')-N(10B)-C(1)	117.0 (2)
C(10A)-N(10B)-C(1)	126.0 (2)
N(10B)-C(3A')-C(3A)	127.6 (2)
N(10B)-C(3A')-C(5A)	121.2 (2)
C(3A)-C(3A')-C(5A)	111.2 (2)
N(22)-C(21)-C(2)	177.6 (2)
C(32)-C(31)-C(36)	119.4 (2)
C(52)-C(51)-C(56)	120.6 (2)
**Torsion angles**	
C(2)C(3)C(31)C(32)	-120.0 (2)
C(2)C(3)C(31)C(36)	56.8 (3)
C(3A)C(3)C(31)C(32)	56.4 (3)
C(3A)C(3)C(31)C(36)	-126.9 (2)
C(4)N(5)C(51)C(52)	-136.8 (2)
C(4)N(5)C(51)C(56)	42.6 (3)
C(5A)N(5)C(51)C(52)	45.8 (3)
C(5A)N(5)C(51)C(56)	-134.8 (2)
**Dihedral angles**	
Best Plane 1/ Best Plane 2	0.51 (4)
Best Plane 3/ Best Plane 2	0.64 (5)
Best Plane 4/ Best Plane 2	60.13 (6)
Best Plane 5/ Best Plane 2	42.55 (8)
Best Plane 6/ Best Plane 2	4.82 (8)
Best Plane 6/ Best Plane 3	5.23 (9)

Best Plane 1 : C(1)C(2)C(3)C(3A)C(3A')C(4)C(5)C(5A)N(6)C(6A)C(7)C(8)C(9)C(10)C(10A)N(10B)N(11)C(21)N(21)
Best Plane 2 : C(1)C(2)C(3)C(3A)C(3A')C(4)C(5)C(5A)N(6)C(6A)C(7)C(8)C(9)C(10)C(10A)N(10B)
Best Plane 3 : C(3A)C(4)C(5)C(5A)N(6)C(6A)C(7)C(8)C(9)C(10)C(10A)N(10B)C(3A')
Best Plane 4 : C(31)C(32)C(33)C(34)C(35')C(36)
Best Plane 5 : C(51)C(52)C(53)C(54)C(55)C(56)
Best Plane 6 : C(1)C(2)C(3)C(3A)C(3A')N(10B)

Table S1.4.

Hydrogen bond geometry and selected weak interactions (Å,º) in the structure of TAAP_LR at T=130(1) K

	D–H	H…A	D...A	∠DHA
**Intramolecular interactions**				
C(10)-H(10)...N(11)	0.95	2.16	2.795 (2)	123
C(56)-H(56)...O(4)	0.95	2.56	2.955 (2)	105
N(11)-H(11)...Cg7	0.90 (2)	2.54	3.127	124
**Intermolecular interactions**				
C(10)-H(10)...Cg4 (-x+2, -y+1, -z+1)	0.95	3.13	3.761	125
C(55)-H(55)...Cg4 (-x+1, -y, -z+2)	0.95	3.05	3.825	140
Cg2...Cg2 (-x+2, -y, -z+1)			3.626	
Cg6...Cg7 (-x+2, -y+1, -z+1)			3.290	
Cg8...Cg8 (-x+2, -y, -z+1)			3.425	
π–π (-x+2, -y, -z+1)			3.413	85.2*

Cg9...Cg9 (-x+2, -y, -z+1)	3.559	
π–π (-x+2, -y, -z+1)	3.394	72.5*

*The angle between Cg-Cg line and the corresponding plane of π-system

Cg2: π-system of C(3A')C(5A)N(6)C(6A)C(10A)N(10B)

Cg4: π-system of C(31)C(32)C(33)C(34)C(35)C(36)

Cg6: π-system of C(1)C(2)C(3)C(3A)C(3A')N(10B)

Cg7: C(21)≡N(22)

Cg8 for condensed π-system:
C(1)C(2)C(3)C(3A)C(3A')C(4)C(5)C(5A)N(6)C(6A)C(7)C(8)C(9)C(10)C(10A)N(10B)

Cg9 for condensed π-system:
C(1)C(2)C(3)C(3A)C(3A')C(4)C(5)C(5A)N(6)C(6A)C(7)C(8)C(9)C(10)C(10A)N(10B)N(11)C(21)N(22)

Table S1.5.

Hydrogen bond geometry and selected weak interactions (Å,º) in the structure of TAAP_RT at T=293(1) K

	D–H	H…A	D...A	∠DHA
**Intramolecular interactions**				
C(10)-H(10)...N(11)	0.93	2.17	2.797 (3)	124
C(56)-H(56)...O(4)	0.93	2.58	2.964 (3)	105
N(11)-H(11)...Cg7	0.86 (1)	2.51 (2)	3.091 (3)	125 (2)
**Intermolecular interactions**				
C(10)-H(10)...Cg4 (-x+2, -y+1, -z+1)	0.93	3.22	3.835	125
C(55)-H(55)...Cg4 (-x+1, -y, -z+2)	0.93	3.12	3.892	142
Cg2...Cg2 (-x+2, -y, -z+1)			3.632	
Cg6...Cg7 (-x+2, -y+1, -z+1)			3.345	
Cg8...Cg8 (-x+2, -y, -z+1)			3.475	
π–π (-x+2, -y, -z+1)			3.468	86.4*
Cg9...Cg9 (-x+2, -y, -z+1)			3.655	
π–π (-x+2, -y, -z+1)			3.455	71.0*

*The angle between Cg -Cg line and the corresponding plane of π-system

Cg2: π-system of C(3A')C(5A)N(6)C(6A)C(10A)N(10B)

Cg4: π-system of C(31)C(32)C(33)C(34)C(35)C(36)

Cg6: π-system of C(1)C(2)C(3)C(3A)C(3A')N(10B)

Cg7: C(21)≡N(22)

Cg8 for condensed π-system:
C(1)C(2)C(3)C(3A)C(3A')C(4)C(5)C(5A)N(6)C(6A)C(7)C(8)C(9)C(10)C(10A)N(10B)

Cg9 for condensed π-system:
C(1)C(2)C(3)C(3A)C(3A')C(4)C(5)C(5A)N(6)C(6A)C(7)C(8)C(9)C(10)C(10A)N(10B)N(11)C(21)N(22)
with π–π responsible for the formation of dimers

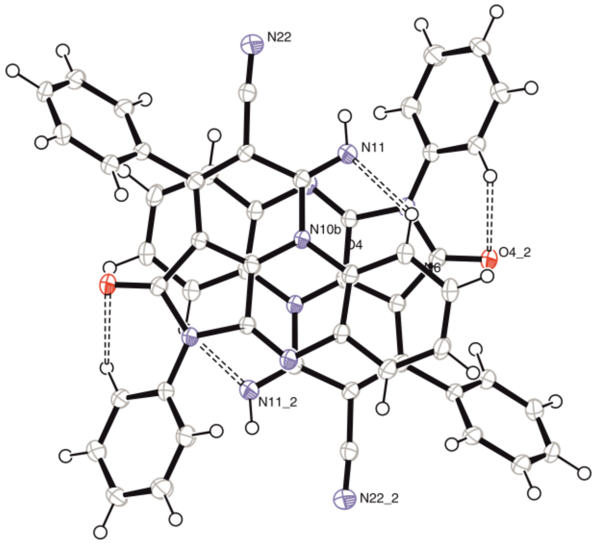

Figure S1.1. Two molecules of TAAP_LT related by the centre of symmetry. The non-hydrogen atoms are represented as displacement ellipsoids at 30% probability levels.

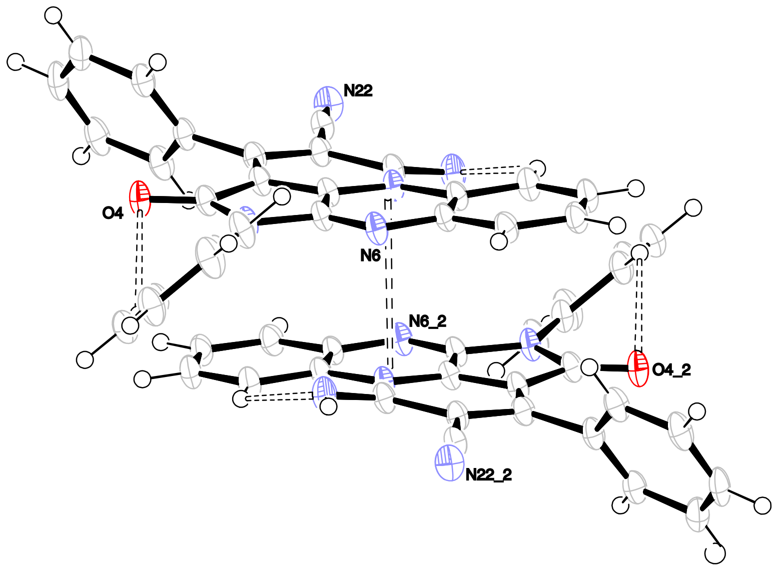

Figure S1.2. Relationship of two neighbouring fluorophores for TAAP_LT with an interplanar distance of 3.413 Å. The non-hydrogen atoms are represented as displacement ellipsoids at 50% probability levels.

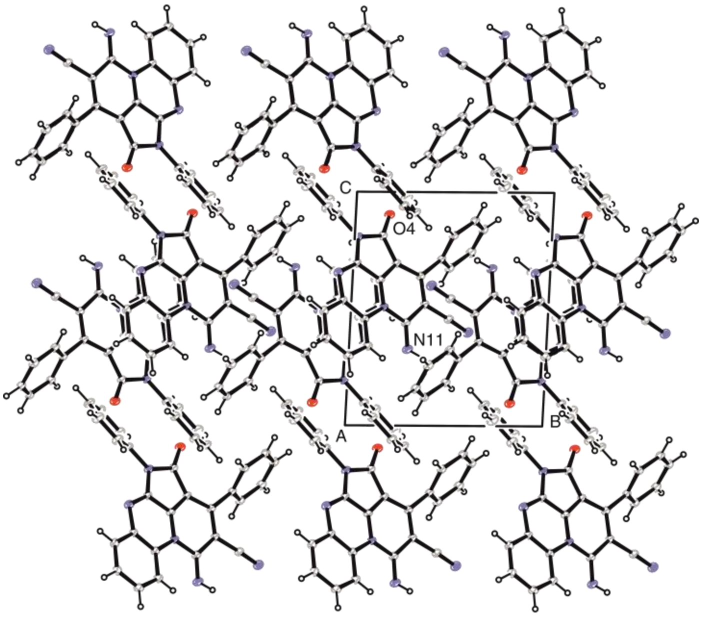

Figure S1.3. Packing of molecule TAAP_LT viewed along [100]. The non-hydrogen atoms are represented as displacement ellipsoids at 30% probability levels.

S1. Non-Covalent Interactions analysis

NCI analysis has been performed for the molecule of TAAP to confirm the existence of the geometrically predicted intramolecular interactions responsible for the decreased structural flexibility. NCI (Non-Covalent Interactions) method utilizes the reduced gradient of electron density s(r) to visualize inter and intramolecular interactions.

$$s(\boldsymbol{r}) = \frac{|\nabla\rho(\boldsymbol{r})|}{2(3\pi)^{1/3}\rho(\boldsymbol{r})^{4/3}}$$

To classify those interactions as favorable or unfavorable we multiply the electron density by the sign of second Hessian eigenvalue ($sign\lambda_2$). Strong and attractive interactions are those with ρ(r)>0 and $\lambda_2 < 0$, weak interactions ρ(r)≈0 and $\lambda_2 \approx 0$ and strong and repulsive interactions are with ρ(r)>0 and $\lambda_2 > 0$. Non-covalent interactions can be visualized as isosurfaces, where small red/blue disc-shaped regions represent strong repulsive/attractive interactions and broad, green and usually irregular surfaces refer to weak interactions. The analysis of interactions was performed via NCIPLOT program (Contreras-Garciá, J.; Johnson, E.R.; Keinan, S.; Chaudret, R.; Piquemal, J-P.; Beratan, D.N., Yang, W. *J. Chem. Theory Comput* 2011, **7**, 625-632.) on the density obtained from DFT calculations using DFT/B3LYP/6-311G**(2d,2p), M. J. Frisch et al., GAUSSIAN 09 (Revision A.1), Gaussian, Inc., Wallingford, CT, 2000) and the results are visible in Figure 2c in main text.

Table S2.1.

Properties of the Bond Critical Points for the intermolecular interactions: $\rho(\mathbf{r})$ – charge density, Laplacian – $\nabla^2\rho$(r) R_{ij} – internuclear separations (Å), d_1, d_2 – distance between BCPs and atom 1, 2 respectively (Å), $V(\mathbf{r})$, $G(\mathbf{r})$ and $E(\mathbf{r})$ local kinetic, local potential and local energy density, respectively. All values except R_{ij} and d_1, d_2 in a.u.

	ρ(r)	$\nabla^2\rho$(r)	R_{ij}	d_1	d_2	V(r)	G(r)	E(r)	|V(r)|/G(r)	E(r)/ρ(r)
H10A...N11A	0.025	0.093	2.093	0.802	1.250	-0.018	0.021	0.003	0.863	0.111
H56A...O4A	0.012	0.044	2.514	1.153	1.435	-0.008	0.010	0.002	0.836	0.133
H52A...N6A	0.009	0.034	2.639	1.156	1.435	-0.006	0.007	0.001	0.789	0.158
N11A...N6B	0.008	0.024	3.184	1.638	1.564	-0.004	0.005	0.001	0.828	0.111
C32A...O4A	0.007	0.024	3.180	1.638	1.581	-0.004	0.005	0.001	0.815	0.133
C10aB...C3a^{1}A	0.006	0.016	3.392	1.719	1.700	-0.003	0.003	0.001	0.799	0.120
N11A...C51B	0.005	0.016	3.396	1.698	1.708	-0.003	0.003	0.001	0.800	0.129
C8A...H36B	0.004	0.014	3.101	1.330	1.783	-0.002	0.003	0.001	0.722	0.179
C7B...C2A	0.005	0.013	3.586	1.758	1.875	-0.002	0.003	0.001	0.811	0.101
H56A...H10B	0.003	0.012	2.716	1.389	1.348	-0.002	0.002	0.001	0.713	0.197

Ground state dipole moment for the molecule of **TAAP** has been calculated using DFT/B3LYP/6-311G**(2d,2p) and the result is visible on **Figure S26**.

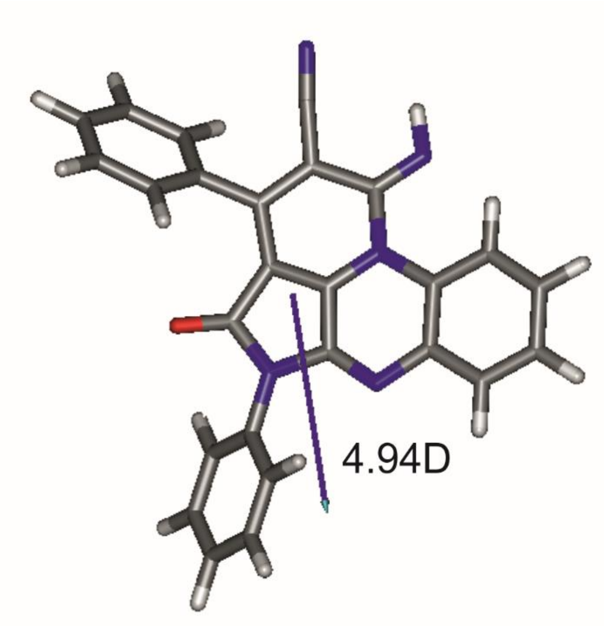

Figure S2.1. The relative orientation of the ground state dipole moment in the molecule of TAAP.

S2. 2D Fluorescence spectra for crystalline solid TAAP and in CH_3CN, benzene-d_6

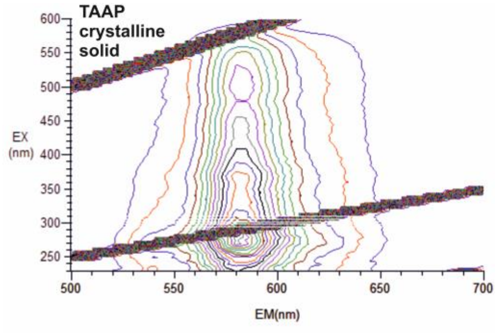

Figure S3.1. 2D Fluorescence spectra for crystalline solid of TAAP**.**

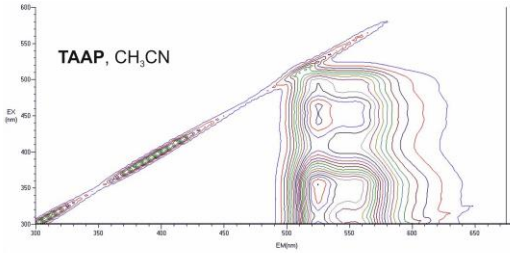

Figure S3.2. 2D Fluorescence spectra for TAAP in CH_3CN**.**

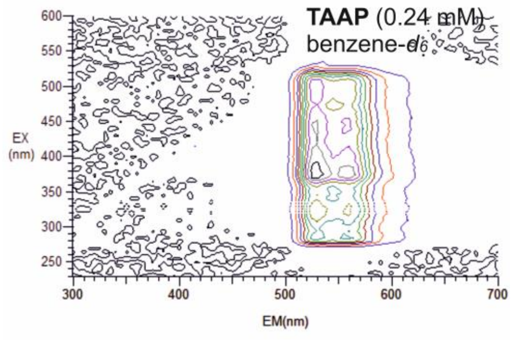

Figure S3.3. 2D Fluorescence spectra for TAAP in benzene -d_6**.**

S3. Determination of fluorescence quantum yield of TAAP in CH_3CN.

Fluorescein in 0.1 M NaOH was used as the reference for determining the fluorescence quantum yield of TAAP in acetonitrile. For this purpose two series of solutions were prepared: one for fluorescein and the other for TAAP. In both cases maximum values of absorbance were applied in the range from 0.04 to 0.1. In this way the inner filter effect was reduced.

All measurements were performed at 20°C. Standard 1.0 cm quartz cells were used for measuring absorbance and fluorescence. Before measurement each sample containing TAAP was degassed by flowing argon through a septum-sealed cell for 25 min.

Absorption spectra were recorded on a Hitachi U2900 spectrophotometer. Fluorescence emission spectra were measured with a fluorescence spectrophotometer Hitachi F7000. Excitation and emission slit widths were set at 5.0 nm. The excitation wavelength used for obtaining fluorescence spectra was 470 nm. From each recorded emission spectrum the solvent spectrum was subtracted and the resulting spectrum was corrected for nonlinearity in instrumental response.

The quantum yield of TAAP in acetonitrile (Φ_i) was determined according the following equation:

$$\Phi_i = \Phi_{std}\left(\frac{m_i}{m_{std}}\right)\left(\frac{n_i^2}{n_{std}^2}\right), \qquad (1)$$

where Φ_{std} is a quantum yield of fluorescein in 0.1 M NaOH ($\Phi_{std} = 0.89$ [Wurth, C.; Grabolle, M.; Pauli, J.; Spieles, M.; Resch-Genger, U. Relative and absolute determination of fluorescence quantum yields of transparent samples. Nat. Protoc. 2013 8, 1535−1550; A guide to recording fluorescence quantum yields. Technical report, Horiba, Jobin Yvon Ltd., 2 Dalston Garden, Stanmore, Middlesex, UK.]), m_i is the slope of a linear fit for the integrated fluorescence intensity of TAAP vs. absorbance, m_{std} is the slope of a linear fit for the integrated fluorescence intensity of fluorescein vs. absorbance (see Fig. S1), n_i is the refractive index of acetonitrile ($n_i = 1.344$), n_{std} is the refractive index of 0.1 M NaOH ($n_{std} = 1.33$). The evaluated value of the quantum yield of TAAP in acetonitrile is thus equal 0.021 (Φ_i = 0.021).

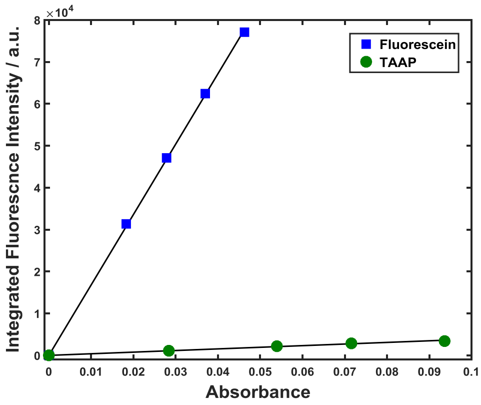

Figure S4.1. Plots of integrated fluorescence intensity versus absorbance at 470 nm for fluorescein in 0.1 M NaOH and TAAP in acetonitrile. The value of m_{std} is equal 1.68 x 10^6 (R^2 = 1.00) and the value of m_i equals 3.87 x 10^4 (R^2 = 1.00).

S4. Absorbance and fluorescence concentration-dependent spectra of TAAP

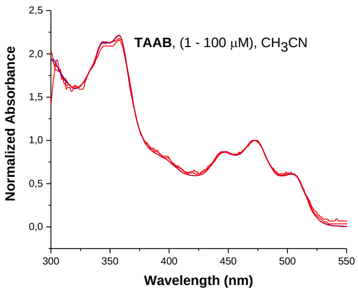

Figure S5.1. Concentration-dependent UV-vis spectra of TAAP in CH_3CN.

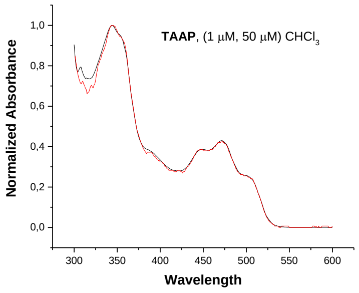

Figure S5.2. Concentration-dependent UV-vis spectra of TAAP in $CHCl_3$.

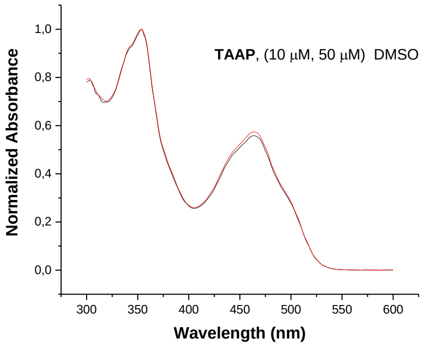

Figure S5.3. Concentration-dependent UV-vis spectra of TAAP in DMSO.

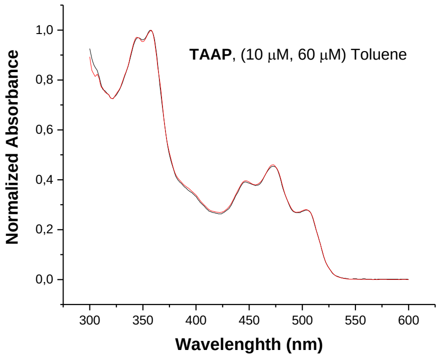

Figure S5.4. Concentration-dependent UV-vis spectra of TAAP in toluene.

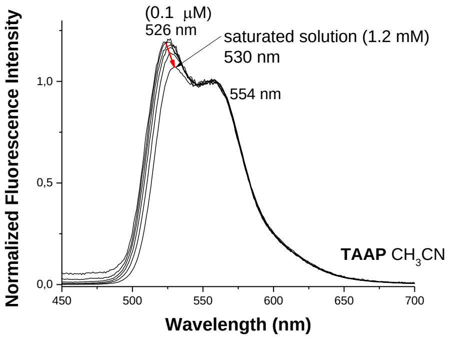

Figure S5.5. Fluorescence spectra of TAAP at concentration range 0.1 μM - 1.2 mM in CH_3CN (λ_{ex} = 440 nm).

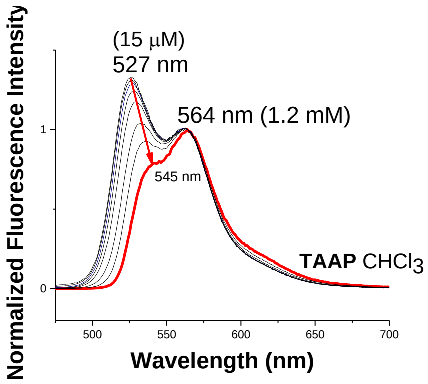

Figure S5.6. Fluorescence spectra of TAAP at concentration range 15 μM - 1.2 mM in $CHCl_3$ (λ_{ex} = 440 nm).

S5. Temperature-dependent fluorescence spectra of TAAP at 296 -361 K.

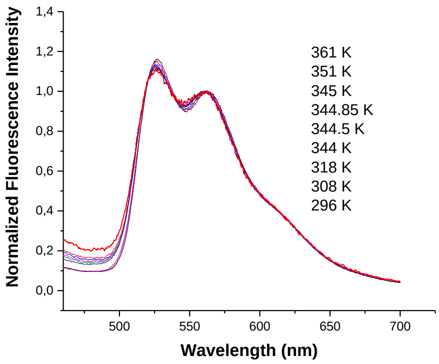

Figure S6.1. Temperature-dependent fluorescence spectra of TAAP at 296 -361 K (λ_{ex} = 440 nm).

S6. Correction of fluorescence intensity for the inner filter effects for TAAP (5 µM, 10 µM, 50 µM, and 100 µM in acetonitrile). The corrected data are drawn in red

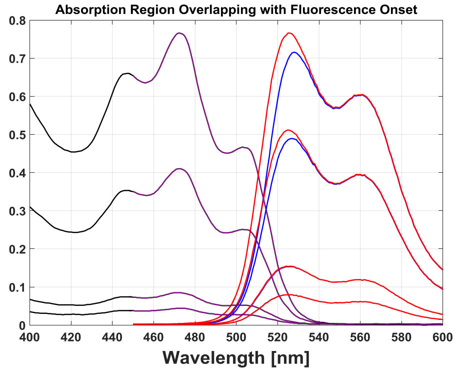

Figure S7.1. UV-vis spectra and fluorescence spectra before and after correction.

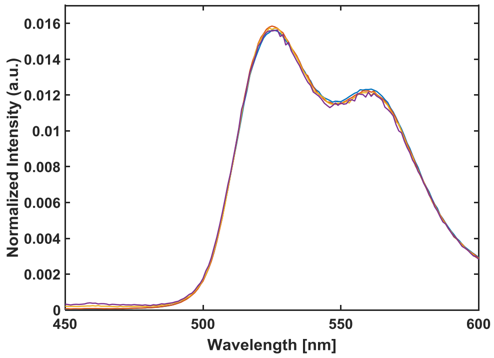

Figure S7.2. Normalized fluorescence spectra after correction.

S7. DFT calculations

B3LYP relaxed coordinates of the dimer in gas phase, followed by: centers of gravity & permanent dipoles of the monomers in the dimer; TDDFT transition dipole moments in atomic units.

```
dimer-relaxed.xyz
C        -1.36714        2.06402        1.47060
C        -2.80635        1.99046        1.10919
C        -3.52303        0.82715        0.85336
C        -2.77332       -0.37742        0.97268
C        -3.07200       -1.79771        0.67829
O        -4.10620       -2.31808        0.31234
N        -1.84695       -2.51791        0.90881
C        -0.86122       -1.63739        1.31911
N         0.36685       -1.89018        1.62719
C         1.13032       -0.78558        1.97932
C         2.46612       -1.04192        2.33402
H         2.78194       -2.07888        2.31919
C         3.33273       -0.01534        2.66241
H         4.36846       -0.23034        2.90076
C         2.86286        1.30306        2.65415
H         3.53509        2.11955        2.89572
C         1.54568        1.59859        2.32427
H         1.18805        2.61507        2.30564
C         0.65669        0.56683        1.97651
N        -0.70707        0.78139        1.61117
C        -1.44843       -0.31682        1.32996
N        -0.68970        3.12790        1.66453
H        -1.25147        3.96252        1.50700
C        -3.43176        3.27129        1.06599
N        -3.87107        4.35025        1.04343
C        -4.95653        0.82818        0.48250
C        -5.84129       -0.07167        1.09723
H        -5.46820       -0.77301        1.83319
C        -7.19303       -0.06927        0.76457
H        -7.86604       -0.76636        1.25379
C        -7.67823        0.81684       -0.19896
H        -8.73160        0.81126       -0.46279
C        -6.80469        1.70796       -0.82464
H        -7.17280        2.39781       -1.57771
C        -5.45467        1.71861       -0.48302
H        -4.78301        2.41034       -0.97654
C        -1.66542       -3.91264        0.66930
C        -0.82579       -4.66081        1.50161
H        -0.32501       -4.18458        2.33386
C        -0.61622       -6.01245        1.23206
H         0.04656       -6.58508        1.87359
C        -1.25263       -6.62698        0.15338
H        -1.08701       -7.68017       -0.05137
C        -2.10393       -5.87756       -0.66019
H        -2.60559       -6.34539       -1.50185
C        -2.31032       -4.52264       -0.41311
H        -2.96678       -3.94192       -1.04525
C         1.36673       -2.06391       -1.47037
C         2.80595       -1.99056       -1.10891
C         3.52280       -0.82733       -0.85312
C         2.77323        0.37733       -0.97241
```

```
C          3.07211        1.79758       -0.67803
O          4.10635        2.31780       -0.31198
N          1.84721        2.51797       -0.90874
C          0.86135        1.63757       -1.31901
N         -0.36665        1.89056       -1.62722
C         -1.13029        0.78606       -1.97930
C         -2.46602        1.04262       -2.33412
H         -2.78157        2.07967       -2.31962
C         -3.33282        0.01614       -2.66237
H         -4.36849        0.23130       -2.90085
C         -2.86321       -1.30235       -2.65385
H         -3.53561       -2.11875       -2.89523
C         -1.54609       -1.59808       -2.32392
H         -1.18861       -2.61461       -2.30516
C         -0.65690       -0.56643       -1.97631
N          0.70683       -0.78120       -1.61095
C          1.44835        0.31691       -1.32974
N          0.68919       -3.12770       -1.66441
H          1.25083       -3.96241       -1.50688
C          3.43116       -3.27148       -1.06568
N          3.87028       -4.35051       -1.04307
C          4.95636       -0.82858       -0.48252
C          5.84109        0.07116       -1.09749
H          5.46787        0.77264       -1.83324
C          7.19295        0.06844       -0.76535
H          7.86593        0.76543       -1.25476
C          7.67834       -0.81788        0.19789
H          8.73182       -0.81258        0.46129
C          6.80485       -1.70885        0.82384
H          7.17309       -2.39882        1.57673
C          5.45469       -1.71917        0.48275
H          4.78308       -2.41073        0.97661
C          1.66583        3.91272       -0.66927
C          0.82656        4.66101       -1.50182
H          0.32595        4.18483       -2.33421
C          0.61714        6.01269       -1.23237
H         -0.04537        6.58541       -1.87411
C          1.25336        6.62713       -0.15353
H          1.08787        7.68036        0.05116
C          2.10430        5.87758        0.66031
H          2.60579        6.34535        1.50210
C          2.31052        4.52263        0.41331
H          2.96668        3.94180        1.04567
X -2.03004  0.61450  -1.03441   # center of mass monomer1
X  2.03004 -0.61450   1.03441   # center of mass monomer2
X -2.03004  0.61450  -1.03441     2.156  3.0061 -0.2149     # permanent dipole (D) monomer1
X  2.03004 -0.61450   1.03441    -2.156 -3.0061  0.2149     # permanent dipole (D) momoner2
X 0 0 0      0.4199     -0.2629     -0.1559  # S1 trans dipole in atomic units (e*bohr)
X 0 0 0     -0.7800      0.6723     -0.1591  # S2 trans dipole in atomic units (e*bohr)
X 0 0 0     -0.0843      0.1798      0.1549..# S3   "     "
X 0 0 0      1.6240      0.1785      0.2787  # S4   "     "
X 0 0 0      0.4383     -0.8781      0.0124  # S5   "     "
X 0 0 0     -0.4199     -0.2598     -0.1094  # S6   "     "
X 0 0 0     -0.2981      0.0565     -0.0266  # S7   "     "
X 0 0 0      0.2458     -0.0777     -0.0284  # S8   "     "
X 0 0 0     -0.1702     -0.0407      0.0708  # S9   "     "
X 0 0 0      0.0427      0.1309     -0.0166  # S10  "     "
```

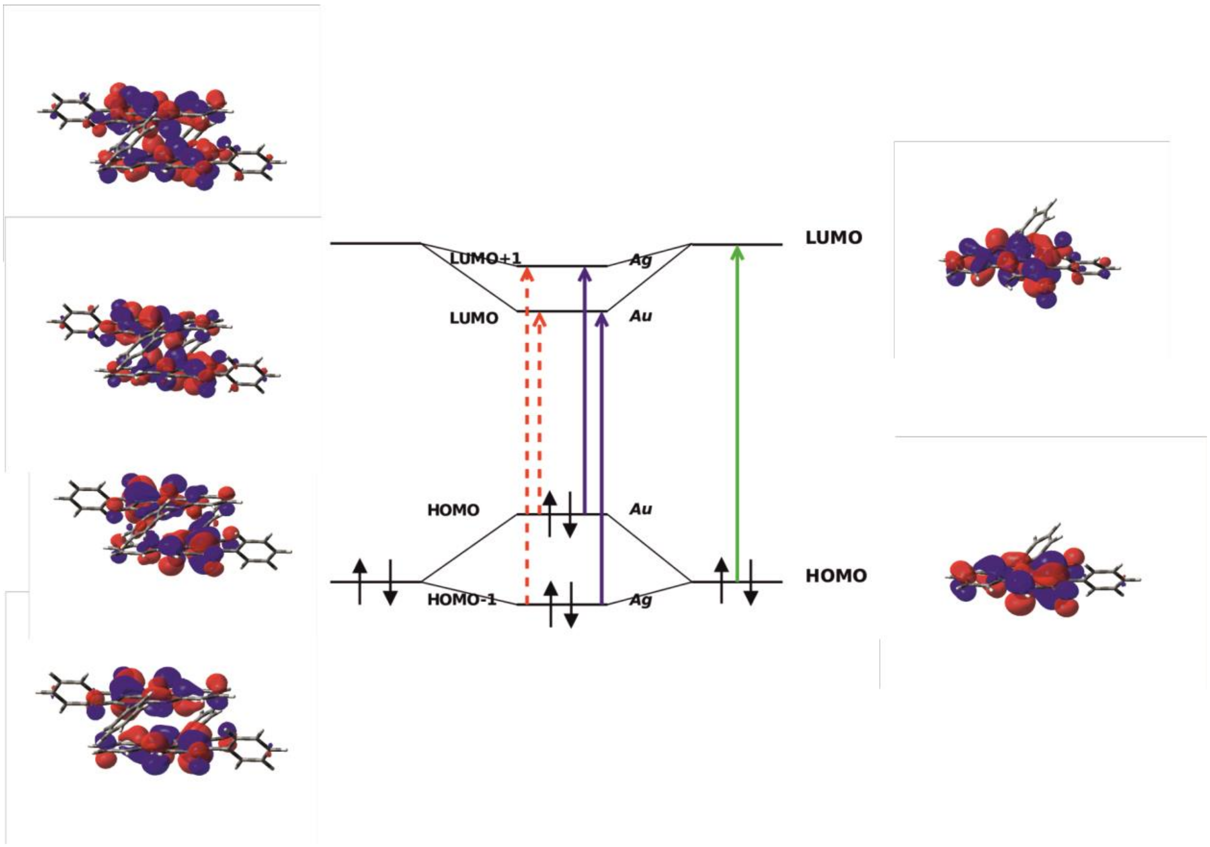

Figure S8.1. Jablonsky diagram for monomer and dimer of TAAP with the frontier orbitals of the monomer HOMO and LUMO, and the frontier orbitals of the dimer: HOMO-1 with A_g symmetry, HOMO with A_u symmetry, LUMO with A_u symmetry, and LUMO+1 with A_g symmetry. The symmetry permitted transitions for the dimer are marked by blue arrows. The symmetry forbidden transitions are indicated by red dashed arrows. The allowed transition for the monomer is marked by green arrow.

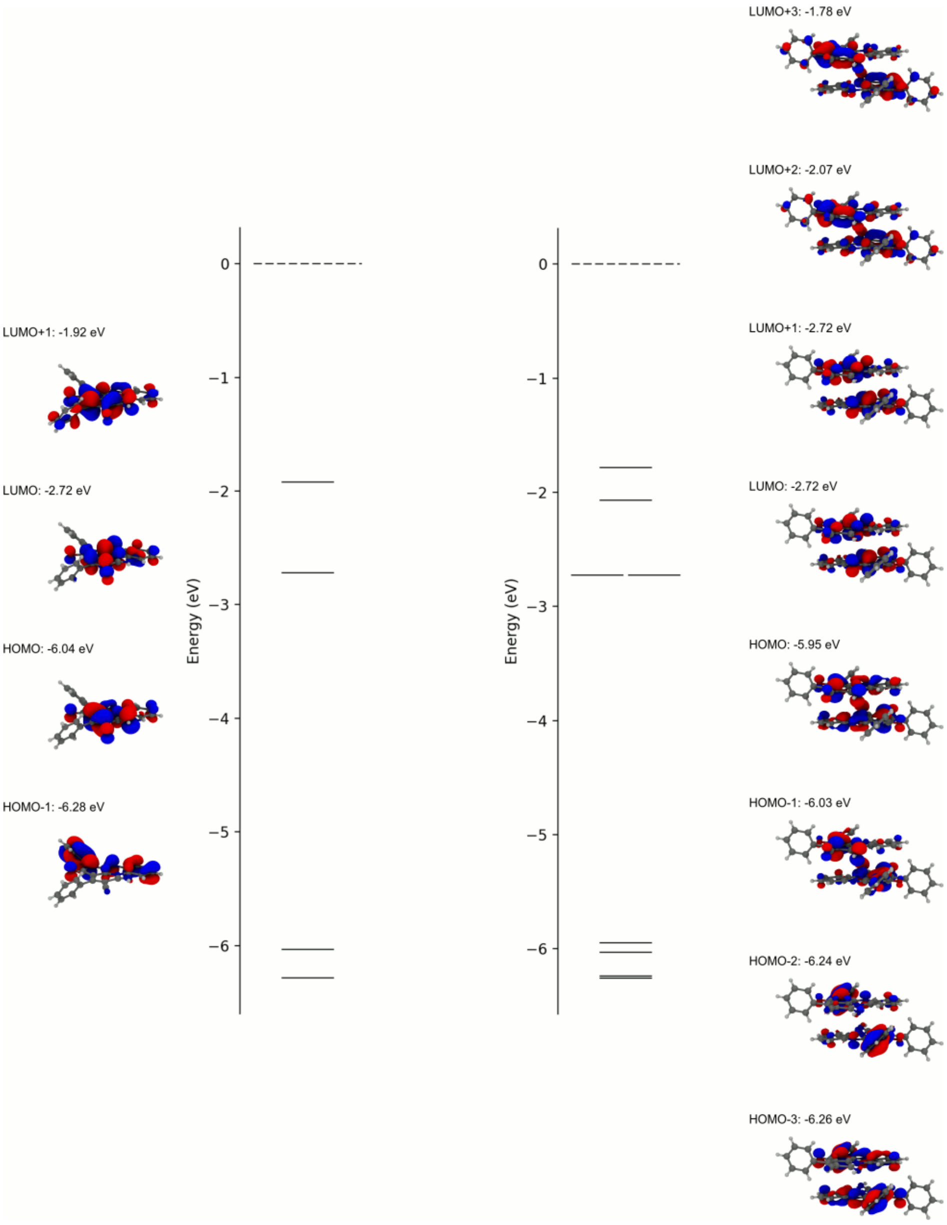

Figure S8.2. Molecular orbitals and energies for the TAAP monomer (left) and dimer (right). The dimer orbital have the following symmetry: HOMO–1 and LUMO are ungerade (A_u), HOMO and LUMO+1 are gerade (A_g). Positive isovalue is blue, negative isovalue is red.

S8. 1D Fluorescence spectra of TAAP and ^{1}H NMR data of TAAP measured at the same concentrations 0.24 mM and 0.1 M in benzene-d_6

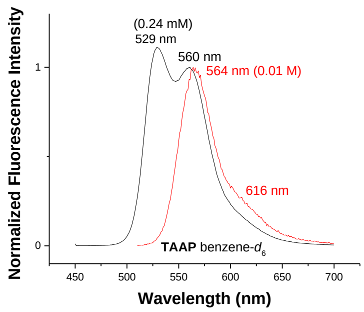

Figure S9.1. 1D Fluorescence spectra of TAAP in different concentration (0.24 mM and 0.01M in benzene-d_6, λ_{ex} = 440 nm).

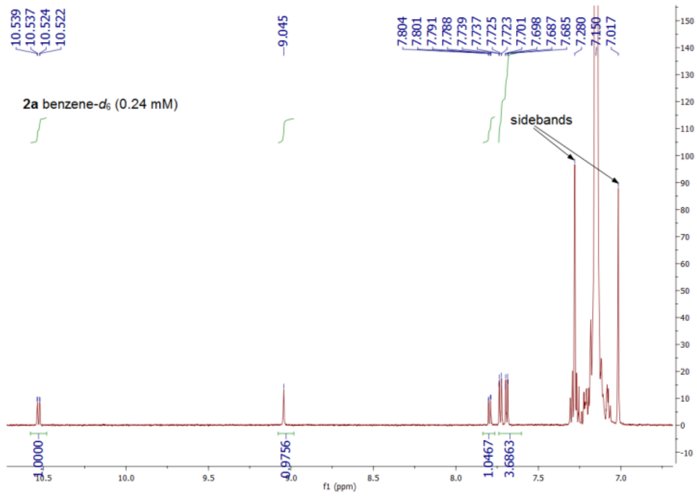

Figure S9.2. ^{1}H NMR data of TAAP measured at the same concentrations 0.24 mM in benzene-d_6.

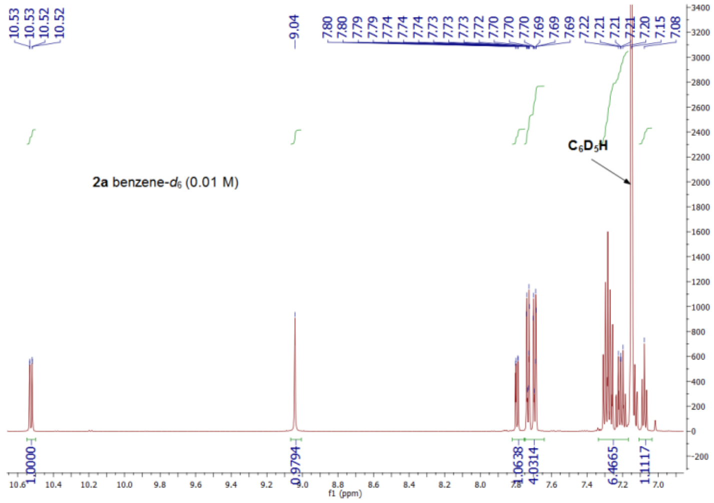

Figure S9.3. ^{1}H NMR data of TAAP measured at the same concentrations 0.01 M in benzene-d_6.

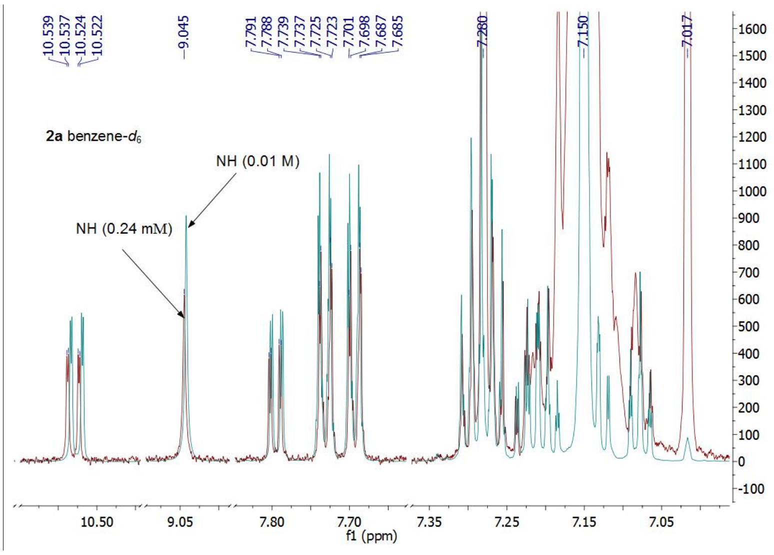

Figure S9.4. Superimposed ^{1}H NMR data of TAAP measured at concentration 0.01 M and .0,24 mM in benzene-d_6.

S9. 2D NMR data for TAAP in THF-d_8

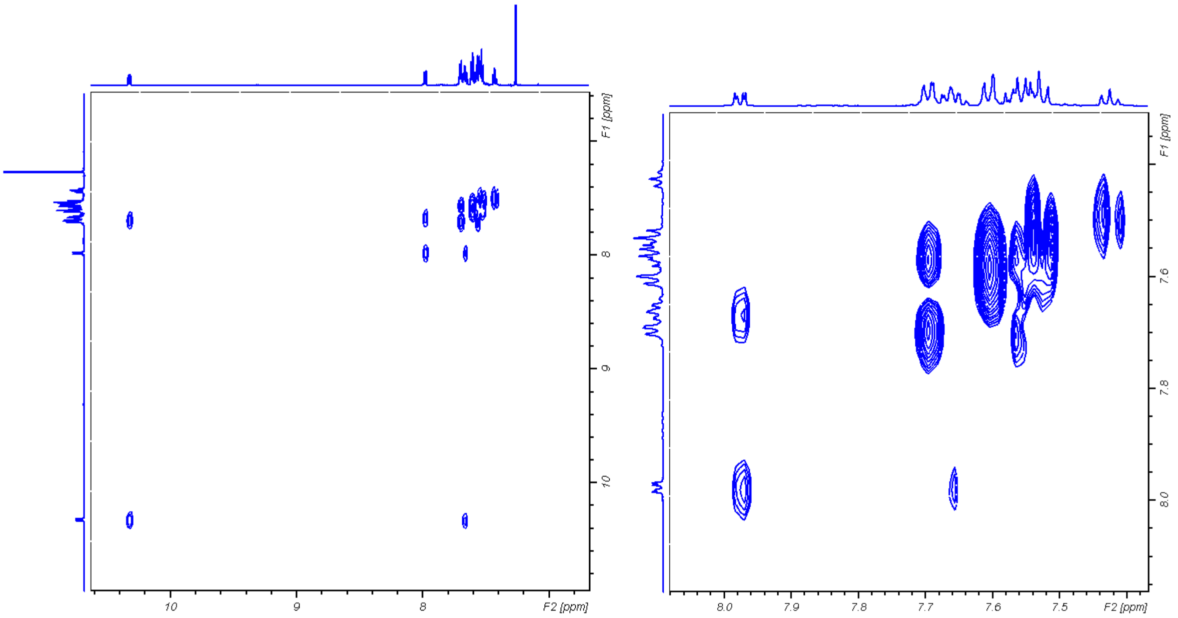

Figure S10.1. ^{1}H-^{1}H-COSY.

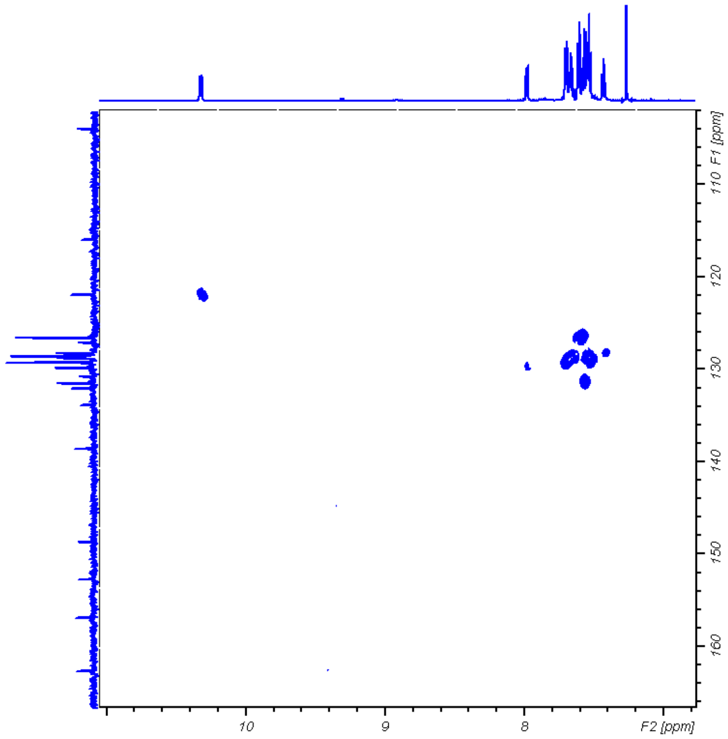

Figure S10.2. HSQC.

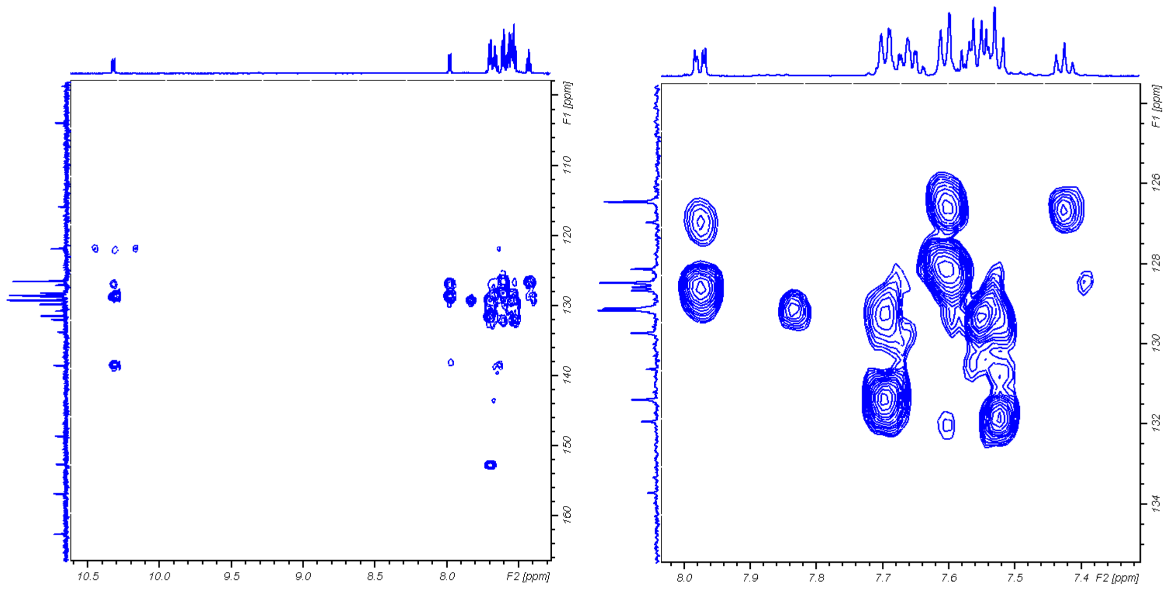

Figure S10.3. HMBC.

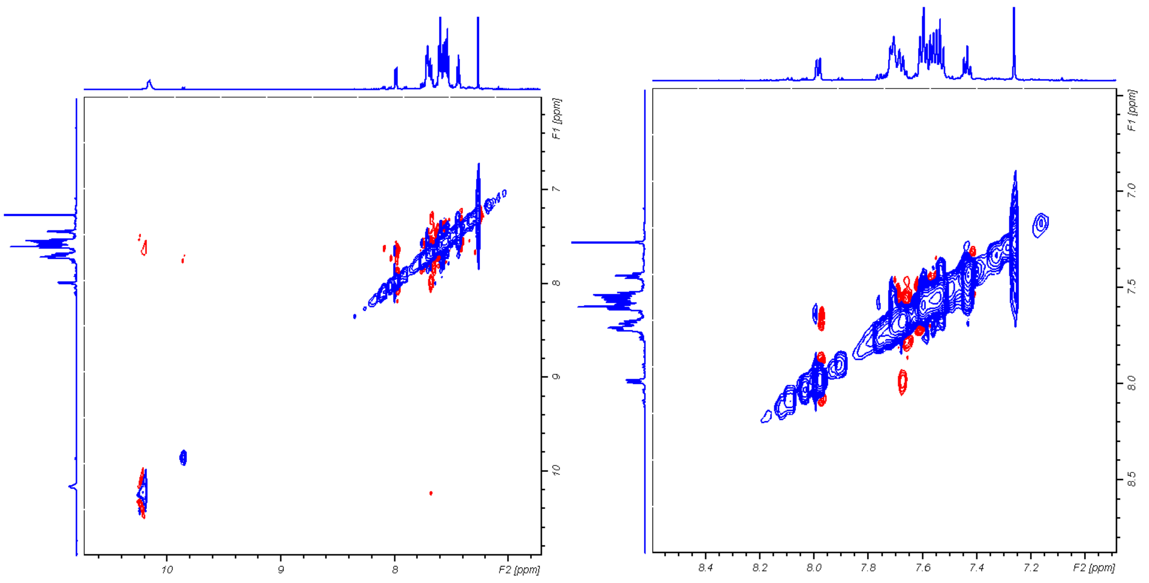

Figure S10.4. ROESY.

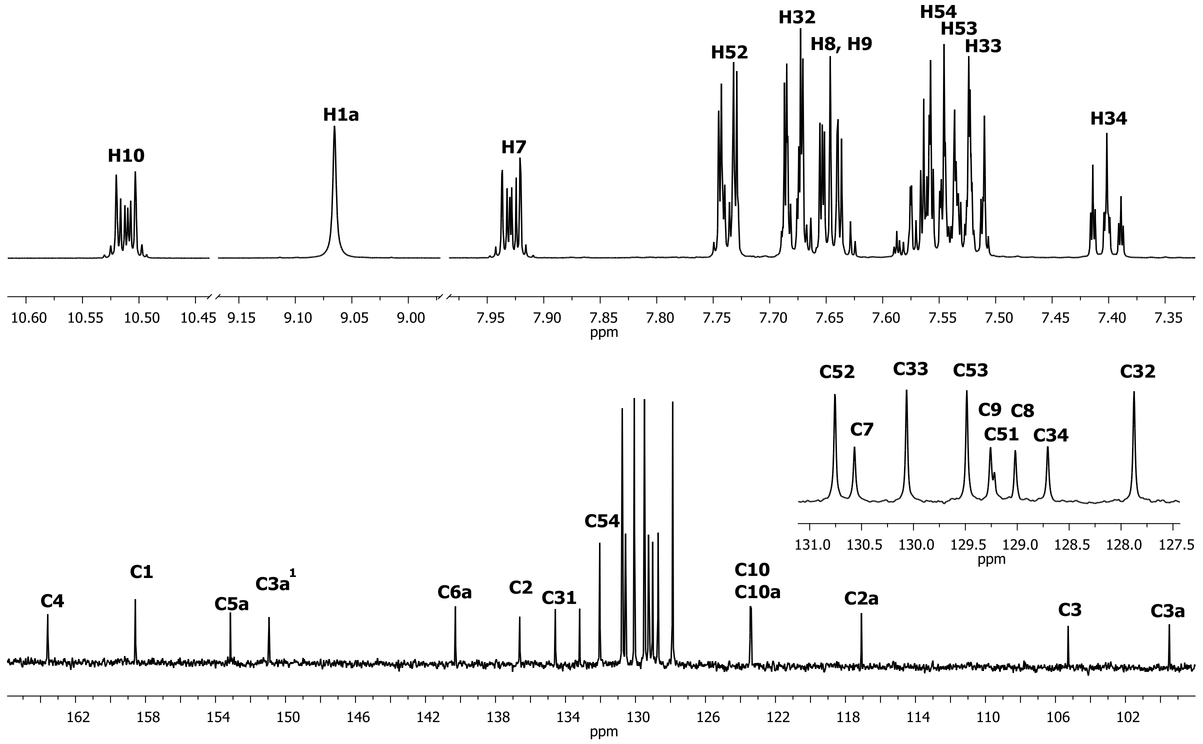

Figure S10.5. ^{1}H and ^{13}C NMR data for TAAP.

S10. Determination of experimental permanent dipole moment

The measurement was performed in 1,4-dioxane saturated solution at 300 K. The dielectric constants of the solutions was measured by a Dipole Meter AGILENT E4980A using condenser cell 16452A. The refractive indices was determined by the use of a Refractometer ATA60 RX5000CX Coubest. The density was measured by Densitometer Mettler Toledo 30PX. The reference liquid was 1,4-dioxane with ε_1 = 2.22.

The dipole moment of TAAP molecule in 1,4-dioxane saturated solution was determined according the following equation:

$$(\boldsymbol{\mu})^2 = \frac{27\boldsymbol{kT}}{4\pi \boldsymbol{N_A}} \frac{\boldsymbol{M_2}}{\boldsymbol{d_1}(\boldsymbol{\varepsilon_1} + 2)^2} \left[\frac{(\boldsymbol{\varepsilon_{12}} - \boldsymbol{\varepsilon_1})}{\boldsymbol{\omega_2}} - \frac{(\eta_{12}^2 - \eta_1^2)}{\boldsymbol{\omega_2}} \right]_{\omega_2 \to 0}$$

where $\boldsymbol{\mu}$ - dipole moment, $\boldsymbol{N_A}$ - Avogadro's number, $\boldsymbol{k}$ - Boltzmann constant, $\boldsymbol{M_2}$ - molar mass of TAAP, ε_1 - dielectric constant of 1,4-dioxane, $\boldsymbol{\varepsilon_{12}}$ - dielectric constant of solution, η_1 - refractive index of 1,4-dioxane, η_{12}, - refractive index of solution, $\boldsymbol{d_1}$ - density of 1,4-dioxane, $\boldsymbol{\omega_2}$ - weight fraction.

S11. Details of Clausius-Mossotti model for optical absorption of molecular solids

S12.1. Introduction

The Clausius-Mossotti (CM) (O. F. Mossotti, *Mem. di mathem. e fsica in Modena*, 24 11, (1850), 49; R. Clausius, *Die mechanische U'grmetheorie*, 2, (1879), p. 62).) equation relates the dielectric constant of a material to the polarizability of its constituents. For example, it relates the dielectric constant of a molecular solid to the polarizability of the molecules. Derivations of the CM equation can be found on most textbooks and many references, such as Ref. (J. H. Hannay, *Eur. J. Phys*. 4, (1983) 131).

Using CGS units, and extending to the frequency dependent response, the molecular polarizability is given by:

$$N\alpha(\omega) = \frac{3}{4\pi}\frac{\epsilon(\omega)-1}{\epsilon(\omega)+2} \qquad (1)$$

where N is the molecular number density in cm ^{3} and is the polarizability in cm^{3}. is related to the \excitation volume".

Then, the CM relation is:

$$\epsilon(\omega) = 1 + \frac{4\pi N\alpha(\omega)}{1-\frac{4\pi}{3}N\alpha(\omega)} \qquad (2)$$

This equation is valid in principle only for cubic systems, but it's a good approximation to the dielectric function for isotropic systems. Note that optical absorption is related to $\epsilon_2 \equiv \mathrm{Im}\,\epsilon$, the imaginary part of the dielectric function. When $N\alpha \leq. 1$ the solid absorption energy ω is markedly shifted from the absorption energy of the free molecule, as an effect of the collective response of all dipoles in the crystal.

S12.2. Molecular polarizability

We assume that the molecular polarizability as a superposition of Lorentian functions, with a small broadening $\gamma \ll 1$:

$$\epsilon(\omega) = \frac{e^2}{m}\sum_k \frac{f_k}{\omega_\kappa^2 - \omega^2 - i\omega\Upsilon} \qquad (3)$$

where the index κ runs over electronic excitations of energy ω_κ and oscillator strength f_κ. Note that Υ is not meant to reproduce vibronic and temperature effects. To take those into account, it is necessary to generate a gaussian of excitations centered around each electronic transition.

The Lorentian form fulfills the Kramers-Krönig relations and after some algebra it leads to the usual form for the imaginary part of the molecular polarizability, in the limit $\Upsilon \to 0$:[1]

$$\alpha_2(\omega) \equiv \mathrm{Im}\alpha(\omega) = \frac{\pi e^2}{2m\omega}\sum_\kappa f_\kappa \delta(\omega \pm \omega_\kappa) \qquad (5)$$

S12.3. Outline of the calculation

Excitation energies ω_k and oscillator strengths f_k are obtained from a TDDFT calculation on an isolated molecular unit (i.e. the dimer in vacuum at the crystal geometry). Next, the complex molecular polarizability $\alpha(\omega)$ is calculated with Υ= 0:002 eV on a very ne energy grid. Then the complex dielectric function is calculated from the CM equation, and convoluted with a gaussian function of width 0.1 - 0.15 eV, to simulate experimental broadening.

The refractive index η and extinction coefficient are obtained by solving the system of equations:

$$\begin{aligned} \mathrm{Re}\,\epsilon(\omega) &= \eta^2 - \kappa^2 \\ \mathrm{Im}\,\epsilon(\omega) &= 2\eta\kappa \end{aligned} \qquad (6)$$

Finallty, the absorbance is calculated as:

$$\eta(\omega) = \frac{2\kappa\omega}{c} \qquad (7)$$

where c is the speed of light.

[1]We used the Plemlj relation:

$$\frac{\omega\gamma}{(e_\kappa^2 - e^2)^2 + \omega^2\gamma^2} \cong \pi\delta(\omega_\kappa^2 - \omega^2)$$

and the following property of the $\delta(x)$ function:

$$\delta\big(F(x)\big) = \sum_\lambda \frac{\delta(x - x_\lambda)}{|F'(x_\lambda)|} \qquad (4)$$

where x_λ are simple zeros of $F(x)$.
